# Blimp-1 benefits gut-homing regulatory T cells by maintaining migration/suppressive function in autoimmune diabetes-prone mice

**DOI:** 10.1016/j.ebiom.2025.106002

**Published:** 2025-11-05

**Authors:** Yi-Wen Tsai, Yu-Wen Liu, Chao-Yuan Hsu, Shin-Huei Fu, Ming-Wei Chien, Jia-Ling Dong, Chi-Chin Sun, Chien-Tzung Chen, Huey-Kang Sytwu

**Affiliations:** a Graduate Institute of Medical Sciences, National Defense Medical University, No. 161, Section 6, Min Chuan East Road, Neihu, Taipei, 114, Taiwan; b Department of Family Medicine, Chang Gung Memorial Hospital, Linkou, No. 5, Fuxing St., Guishan Dist., Taoyuan City, 333, Taiwan; c Department of Family Medicine, New Taipei Municipal Tucheng Hospital (Bulit and Operated by Chang Gung Medical Foundation), No. 6, Section 2, Jincheng Road, Tucheng District, New Taipei City, 236, Taiwan; d School of Medicine, College of Medicine, Chang-Gung University, No. 259, Wenhua 1st Road, Guishan District, Taoyuan City, 333, Taiwan; e Graduate Institute of Microbiology and Immunology, National Defense Medical University, No. 161, Section 6, Min Chuan East Road, Neihu, Taipei, 114, Taiwan; f National Institute of Infectious Diseases and Vaccinology, National Health Research Institutes, No. 35, Keyan Road, Zhunan, Miaoli, 350, Taiwan; g Graduate Institute of Life Sciences, National Defense Medical University, No. 161, Section 6, Min Chuan East Road, Neihu, Taipei, 114, Taiwan; h Department of Ophthalmology, Chang Gung Memorial Hospital, Keelung, No. 222, Maijin Road, Keelung, 204, Taiwan; i Department of Plastic and Reconstruction Surgery, Chang Gung Memorial Hospital, Linkou, No. 5, Fuxing St., Guishan Dist., Taoyuan City, 333, Taiwan

**Keywords:** Blimp-1, Gut-homing regulatory T cells, CCR6^+^RORγt^+^ Th17-like Treg cells, T-cell receptor signaling strength, Crohn's disease, Type 1 diabetes

## Abstract

**Background:**

Genome-wide association studies (GWAS) have shown that Crohn's disease (CD) and type 1 diabetes (T1D) are the top 2 diseases with the highest genetic risk variants and share several susceptible loci. Since both CD and T1D are T cell-mediated diseases, we hypothesise a mechanistic linkage between T-cell homeostasis and a gut–pancreas axis that differentially regulates the immunopathogenesis and development between CD and T1D.

**Methods:**

Using data for 1 million people from a 16-year nationwide population-based databank in Taiwan, we unraveled a higher risk of prevalent inflammatory bowel disease in T1D patients. This observation is supported by our model of T-cell-specific B-lymphocyte-induced maturation protein 1 (Blimp-1) deficiency-induced colitis, in which more severe colitogenesis was observed in diabetes-prone non-obese diabetic (NOD) mice than in non-diabetes-prone C57BL/6 mice.

**Findings:**

Mechanistic investigations revealed that, compared with Tregs in C57BL/6 background, those in Blimp-1-deficient NOD mice exhibited decreased suppressive function, increased TCR signaling strength and impaired intestinal migration, particularly for gut-homing Th17-like Tregs. Strikingly, transgenic augmentation of PEST domain-enriched tyrosine phosphatase (Pep) to downregulate TCR signaling strength reversed exacerbated colitis and increased disease-free percentage of Blimp-1-deficient NOD mice. Adoptive transfer experiments further supported that Pep overexpression restored suppressive function of fragilized Tregs in Blimp-1-deficient NOD mice.

**Interpretation:**

Our results demonstrate that Blimp-1 sustains the suppressive function of gut-homing Tregs and that Pep-based TCR signaling manipulation may serve as a therapeutic target in autoimmune diseases.

**Funding:**

This study was funded by the Ministry of Science and Technology, Taiwan (MOST109-2320-B-400-018-MY3, MOST110-2320-B-400-011-MY3, MOST109-2314-B-182A-149); the 10.13039/100020595National Science and Technology Council, Taiwan (NSTC112-2320-B-400-026-MY3, NSTC113-2320-B-400-019-MY3); the 10.13039/501100010425Tri-Service General Hospital (TSGH-C02-112029, TSGH-C03-113037, TSGH-C01-114028, VTA112-T-1-1, VTA113-T-1-1); and the 10.13039/100012553Chang Gung Memorial Hospital Research Projects (NMRPG2K0021, CMRPG2M0041, CMRPVVM0182, CORPVVN0131, CMRPG2I0071, CMRPG2I0072, CMRPG2I0073, CORPG3P0622).

**Research in context**

**Evidence before this study**

Meta-analysis of genome-wide association studies identified that Crohn's disease and type 1 diabetes shared common susceptible genetic loci, revealing important insights into their etiologic features regulated by common biologic mechanisms. There was a potential epidemiological correlation between these two diseases but a causal relationship remains elusive.

**Added value of this study**

This study integrates epidemiologic analysis with experimental models to uncover the positive correlation between T cell-specific Blimp-1 in autoimmune diabetes and the risk for developing Crohn's disease. Blimp-1 deficiency impairs gut-homing Treg's suppressive function and migration via increased TCR signaling in diabetes-prone NOD mice, compared to non-diabetes-prone C57BL/6 genetic background. Transgenic augmentation of Pep to downregulate TCR signaling strength can significantly reverse exacerbated colitis and restore the suppressive function of these fragile Tregs.

**Implications of all the available evidence**

The translational potential of modulating PEP in Tregs may improve Treg-based immunotherapies and help to prevent autoimmune progression in people with T1D.

## Introduction

The results of genome-wide association studies (GWAS) have suggested a genome-based association between autoimmune diseases and their genetic predisposing factors based on the identification of several susceptible genetic loci,^1^ among which, Crohn's disease and type 1 diabetes (T1D) are the top 2 diseases with the highest numbers of uncovered genes.^1^^,^^2^ Interestingly, several genes, such as *PTPN22*, *IL2RA*, *IL18RAP*, *IL27*, and *BACH2*, have been shown to be highly susceptible in, and shared between, Crohn's disease and T1D.^2^, ^3^, ^4^, ^5^ Considering the genome-based association between Crohn's disease and T1D identified by GWAS, we have verified a potential epidemiological correlation between these 2 diseases in the real world by conducting a nationwide population-based study in Taiwan that included data for 1,000,000 randomly selected people from the 16-year National Health Insurance Research Database (NHIRD) obtained from 1997 to 2013. This national program covers over 99.6% of Taiwan's population because every Taiwanese person who is registered in the census for over 6 months is required to join the National Health Insurance system, the health care and administrative data sets of which are maintained electronically by the National Health Research Institute and National Health Insurance Administration of Taiwan.^6^, ^7^, ^8^ After adjusting for various potential confounding factors, we found a significantly increased risk of inflammatory bowel disease (IBD), especially Crohn's disease (CD), in people with T1D, substantiating the GWAS data about the genetic association between these two diseases. This finding is also supported by a recent published report by Sun et al.^9^ based on Swedish epidemiological analysis.

Because both CD and T1D are T cell–mediated inflammatory and share some genetic predisposing factors diseases,^10^^,^^11^ we hypothesised a shared mechanistic link involving T cell homeostasis and the gut–pancreas axis. Immune tolerance regulation involves critical factors like B-lymphocyte-induced maturation protein 1 (Blimp-1), encoded by *Prdm1*, a master regulator of B- and T-cell differentiation and activation.^12^, ^13^, ^14^ We previously demonstrated that Blimp-1 overexpression in T cells protects against diabetes and MOG_35–55_-induced encephalomyelitis in non-obese diabetic (NOD) mice, whereas its deletion increases Th1/Th17 responses and exacerbates encephalomyelitis.^15^^,^^16^ Surprisingly, instead of more severe diabetes, these T cell-specific Blimp-1 conditional knockout (CKO). NOD mice developed colitis,^17^ consistent with previous reports that deletion of *Prdm1* in T cells causes spontaneous colitis in mixed 129 and C57BL/6 mice.^12^^,^^13^ It is unlikely that Blimp-1 deficiency-mediated colitis is a confounding factor that contributes to the resistance of spontaneous diabetes in NOD mice because the phenotype of autoimmune diabetes could be restored when Blimp-1 CKO. NOD mice are crossed with BDC2.5 T-cell receptor (TCR) transgenic NOD mice. These results indicate that Blimp-1 deficiency-induced colitis and diabetic resistance can be overridden by an islet-specific T cell repertoire, suggesting a critical “switch” control and “nonreciprocal” regulation of Blimp-1 in the pathogenesis of autoimmune diabetes. Based on other findings that the Blimp-1 mRNA expression is greater in Tregs than in conventional T cells (Tconvs) and that Blimp-1 critically controls Treg suppressive function in dextran sodium sulfate-treated C57BL/6 mice,^13^ we explored the immunomodulatory function and underlying mechanisms of Blimp-1 in Tregs in diabetes-prone NOD mice.

Recent studies have highlighted the plasticity of Tregs, which can adopt features of effector T helper (Th) subsets while retaining Foxp3 expression,^18^^,^^19^ and are therefore named Th-like Tregs. For instance, a specific Th17-like Treg subset can secrete IL-17 and express the chemokine receptor 6 (CCR6) and transcription factors, such as retinoic acid-related orphan receptor gamma t (RORγt), but possess the ability to repress the Th17 inflammatory response.^20^, ^21^, ^22^ In a T-cell transfer colitis model, CCR6^−/−^ Tregs with impaired IL-10 production exhibited a defective suppressive function toward colitogenic inflammation in *Rag2*^−/−^ C57BL/6 recipients, implying a critical role of CCR6 in Th17-like Treg function. Additional findings from T lymphocytes in colitis-transfer models and patients with pancreatic ductal adenocarcinoma have shown that Tregs with RORγt coexpression possess both proinflammatory and enhanced immunosuppressive properties, while decreased CD25 expression suggests a phenotypic and functional switch toward the Th17 lineage.^22^^,^^23^ Taken together, the accumulating evidence indicates that a balance between Th-like Tregs and Th cells plays a pivotal role in controlling immune homeostasis and disease progression. Here, we further investigate how these Th17-like Tregs and the chemokine receptor CCR6 affect the immunoregulation of Blimp-1 deficiency-mediated colitis in diabetes-prone NOD mice.

Treg suppressive function is regulated by TCR signaling and costimulatory activation.^24^ A subset of highly self-reactive Tregs with enhanced TCR signaling, characterised by high CD5 and nuclear receptor subfamily 4 group a member 1 (Nur77) expression, loses its ability to prevent colitis in a T-cell transfer model, suggesting an inverse correlation between TCR signaling strength in Tregs and their suppressive function in colitis models.^25^ Of the T1D loci reported to date, we focused on *PTPN22* because GWAS data identify it as a key shared risk gene between CD and T1D.^2^, ^3^, ^4^ *PTPN22* encodes the phosphatase PEP, which inhibits TCR signaling by dephosphorylating Src family kinases.^26^ Notably, the gain-of-function SNP C1858T in *PTPN22* has been associated with a lower risk of CD^4^. Following our previous demonstration that T cell-specific Pep overexpression alleviates insulitis and diabetes in NOD mice,^27^ we further explored whether the transgenic overexpression of Pep to downregulate TCR signaling could restore the impaired suppressive function of Blimp-1-deficient Tregs and subsequently ameliorate colitis in autoimmune diabetes-prone Blimp-1-deficient NOD mice. Finally, the cellular and molecular mechanisms by which Blimp-1 regulates the migration and suppressive function of gut-homing CCR6^+^RORγt^+^ Th17-like Tregs were also investigated.

## Methods

### Study population/National Health Insurance Research Database

**Fig. 1:**
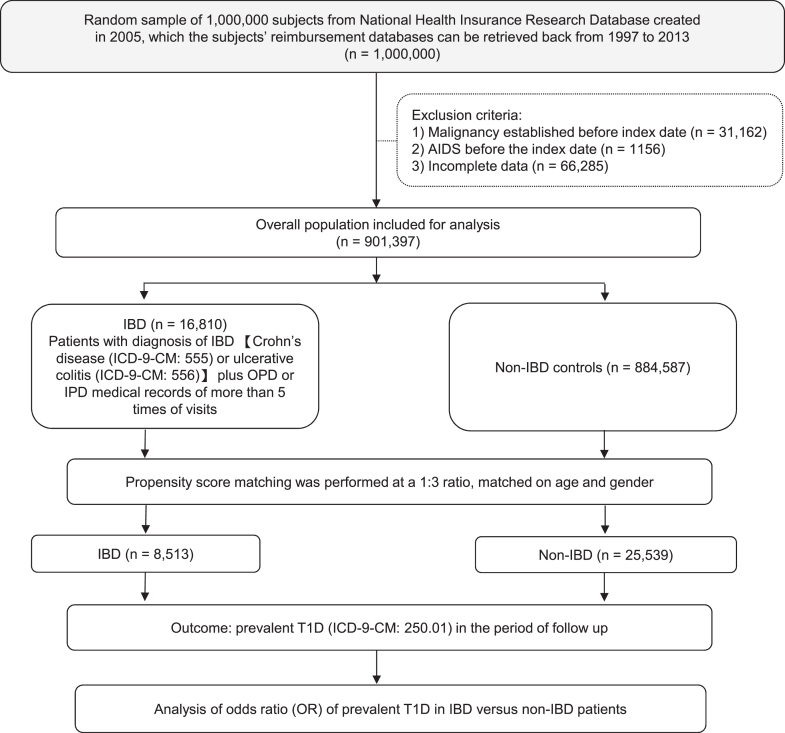
**Flowchart: analysis of the overall risk of prevalent T1D in people with and without IBD using data from a nationwide population-based database in Taiwan.** Abbreviations: AIDS, Acquired Immunodeficiency Syndrome; IBD, inflammatory bowel disease; ICD-9-CM, International Classification of Diseases, Ninth Revision, Clinical Modification; OPD, outpatient department; IPD, inpatient department; T1D, type 1 diabetes.

We analysed data from the Longitudinal Health Insurance Database 2005, a nationally representative dataset composed of 1,000,000 individuals randomly selected from all enrollees in Taiwan's National Health Insurance (NHI) program in 2005 using a stratified random sampling method based on age, sex, and geographic region. Data were extracted and analysed from January 1st, 1997 to December 31st, 2013 to determine the correlation between prevalent IBD (including CD and UC) and T1D, as well as other autoimmune diseases. The National Health Insurance system covers over 99.6% of Taiwan's population, providing de-identified outpatient, emergency, and admission records for research.^6^, ^7^, ^8^ ICD-9-CM codes for IBD were defined as 555.xx for Crohn's disease or 556.xx for UC.^28^^,^^29^ The index date was defined as the date of first IBD diagnosis for cases and the corresponding matched date for non-IBD controls. During initial NHIRD preprocessing, we excluded individuals with malignancy or AIDS, conditions that could potentially affect immune regulation, as well as those with incomplete data, such as missing age, sex, or registry claims records. The final analytic cohort was separated into IBD cases and non-IBD controls. Propensity score matching (PSM) was applied at a 1:3 ratio on age and sex to create a balanced case–control dataset. Following matching, the study population consisted of an IBD group (cases) and a non-IBD group (disease-naïve controls) (Fig. 1). Information on race and ethnicity is not available in the NHIRD, in accordance with local regulations; all data are de-identified and provided in a delinked format to protect patient privacy.

### Statistical analysis

#### Population-based study

Multivariate conditional logistic regression was used to estimate the adjusted ORs for autoimmune diseases in the IBD group compared to the matched non-IBD controls, accounting for matching strata (age and sex) and adjusting for relevant comorbidities such as dyslipidemia, hypertension, ischemic heart disease, and chronic obstructive pulmonary disease. Sex was self-reported by participants. Since all covariates were derived from claims-based diagnostic codes in the NHIRD, no missing data were encountered in the variables included in the conditional logistic regression analysis. T1D was defined as at least two visits to an outpatient clinic and a disease-specific diagnosis code noted by a physician plus insulin-only treatment after diagnosis. A *p* value less than 0.05 was considered significant. All statistical analyses were performed using SAS EG software, version 9.4 (SAS Institute Inc., Cary, NC, USA).

#### Animal experiments

Survival curves were compared using the Mantel–Cox log-rank test. Data are presented as mean ± standard error of the mean (SEM). Normality was assessed by Shapiro–Wilk test. For normally distributed data, two-group comparisons used unpaired two-tailed Student's t-tests, and multiple-group comparisons one-way ANOVA with Tukey's post hoc test; repeated-measures ANOVA with applied for longitudinal data. For non-normal datasets, Mann–Whitney U test and the Kruskal–Wallis test were conducted. Sample sizes were limited by ethical animal use, potentially reducing power to detect small effects. A *p* value < 0.05 was considered statistically significant. All analyses were performed using GraphPad Prism (version 8).

### Mice

NOD/Sytwu (K^d^, D^b^, I-A^g7^, I-E^null^) mice were initially purchased from the Jackson Laboratory (Bar Harbor, ME, USA). NOD.*Cre*^*Lck*^*Prdm1*^*F/F*^ and C57BL/6.*Cre*^*Lck*^*Prdm1*^*F/F*^ mice were established according to our previous report.^16^ Pep-overexpressing Blimp-1 CKO. NOD mice were established by crossing *Lck*-*Cre* promotor-driven *Ptpn22* transgenic NOD mice^27^ with Blimp-1 CKO. NOD mice. All mice were bred and maintained at the Animal Center of the National Defense Medical Center (Taipei, Taiwan) in a specific pathogen-free facility.

Flow cytometric analysis, RNA sequencing (RNA-seq), in vitro Treg suppression assay, migration assay, disease monitoring and histological scoring, and adoptive transfer methods can be found in the Supplemental Methods.

### Assessment of diabetes

The onset of diabetes in NOD mice was monitored weekly by measuring urinary glucose concentration using Chemstrips (Roche, Boehringer Mannheim). Diabetes was defined as glycosuria greater than 500 mg/dL on two consecutive measurements. We used both male and female NOD mice throughout the study. However, diabetes incidence was only performed in female NOD mice because females exhibit a more pronounced autoimmune response in the islets, which is similar to type 1 diabetes. The disease onset of female NOD mice starts at approximately 12 weeks and reaches about 80% by 40 weeks.^30^

### Ethics

The protocol of this nationwide population-based study was approved by the Institutional Review Board of Chang Gung Memorial Hospital (201900566B0). As the study used retrospective data from the National Health Insurance Research Database (NHIRD), which is regulated by the National Health Insurance Administration and only provides de-identified data with all personal identifiers removed, the requirement for initial consent was waived. In line with this, the IRB also approved the waiver of the reconsent process. Mouse experiments were approved and conducted in accordance with institutional guidelines and approved by the National Defense Medical Center Institutional Animal Care and Use Committee. All procedures adhered to the institutionally approved protocol (IACUC0-20-107, IACUC-23-073).

### Role of funders

The funders were not involved in any part of the study design, data collection, data analyses, interpretation, or writing of report.

## Results

### Prevalent IBD is significantly associated with T1D in a nationwide population-based case–control study in Taiwan

To identify any correlation between the prevalence of IBD, including CD or ulcerative colitis (UC), and other autoimmune diseases, we analysed data from the 16-year National Health Insurance Research Database in Taiwan and performed propensity score-matched analysis to divide the patients into an IBD group and a non-IBD (disease-naïve) control group (Fig. 1). People with T1D, systemic lupus erythematosus, psoriasis, rheumatoid arthritis, ankylosing spondylitis and multiple sclerosis were identified in both IBD and control group (Table 1 and Supplemental Table S1). Given that Crohn's disease and T1D are the top two diseases with the highest numbers of uncovered susceptible genes, we utilised multivariate logistic regression to compare the odds ratios and 95% confidence intervals of the associations between the IBD and non-IBD group. After adjusting for potential confounding factors, individuals with T1D had significantly higher odds of having IBD, particularly CD, compared to matched controls (adjusted OR for IBD: 1.54; 95% CI: 1.13–2.09; *p* = 0.006; adjusted OR for CD: 1.46; 95% CI: 1.06–2.00; *p* = 0.021) (Table 2). We also found a higher risk of other prevalent autoimmune diseases in the IBD compared with the non-IBD control group (Supplemental Table S2). Our 16-year nationwide population-based epidemiological case–control analysis demonstrated a significant positive correlation between prevalent IBD and T1D, especially for CD, consistent with previous GWAS findings.

**Table 1:** Demographics and underlying medical conditions in the IBD vs non-IBD groups (1997–2013).

Characteristics	Overall population (Before Match)					Matched on propensity score (After Match)				
	IBD (n = 16,810)		non-IBD (n = 884,587)		SMD	IBD (n = 8513)		non-IBD (n = 25,539)		SMD
	N (Mean)	SD (%)	N (Mean)	SD (%)		N (Mean)	SD (%)	N (Mean)	SD (%)	
**Mean age, years (SD%)**	35.69	23.34	36.33	20.81	−0.029	35.39	23.86	35.39	23.84	0.000
**Age group, n (%)**										
0–10 years	3276	19.47%	108,870	12.31%	0.197	1886	22.15%	5639	22.08%	0.002
10–20 years	2010	11.95%	112,499	12.74%	−0.024	821	9.64%	2468	9.66%	−0.001
20–30 years	181	10.81%	132,904	14.97%	−0.124	825	9.69%	2554	10.00%	−0.010
>30 years	9719	57.77%	530,314	59.98%	−0.045	4981	58.51%	14,878	58.26%	0.005
**Sex, n (%)**										
Male	8552	50.84%	443,434	51.57%	−0.015	4330	50.86%	12,980	50.82%	0.001
Female	8271	49.16%	441,153	48.43%	0.015	4183	49.14%	12,559	49.18%	−0.001
**Comorbidities, n (%)**										
Dyslipidemia	2917	17.35%	132,510	14.98%	0.064	1283	15.07%	2970	11.63%	0.101
Hypertension	3448	20.51%	155,629	17.59%	0.074	1666	19.57%	3737	14.63%	0.131
Ischemic heart disease	2251	13.39%	86,036	9.73%	0.115	1086	12.76%	2030	7.95%	0.158
COPD	2637	15.69%	102,562	11.59%	0.119	1277	15.00%	2405	9.42%	0.171
Chronic kidney disease	1599	9.51%	66,093	7.47%	0.073	743	8.73%	1483	5.81%	0.113
Liver cirrhosis	4118	24.50%	184,848	20.90%	0.086	1972	23.16%	3805	14.90%	0.212
**T1D, n (%)**	132	0.79%	5266	0.60%	0.023	73	0.86%	141	0.55%	0.037

Abbreviations: IBD, inflammatory bowel disease; SMD, standardized mean difference; COPD, chronic obstructive pulmonary disease; T1D, type 1 diabetes.

SMDs are presented to evaluate covariate balance before and after propensity score matching, which included age and sex. An SMD <0.1 indicates adequate balance, whereas an SMD >0.1 suggests potential imbalance.

**Table 2:** Conditional logistic regression analysis of the adjusted ORs for IBD in people with T1D (1997–2013).

Parameter	Crude OR^a^	95% confidence interval	*p* value	Adjusted OR^b^	95% confidence interval	*p* value
IBD (UC or Crohn's disease)	1.57	1.18–2.08	0.002	1.54	1.13–2.09	0.006
Crohn's disease	1.52	1.13–2.05	0.005	1.46	1.06–2.00	0.021
UC	2.25	0.78–6.5	0.133	2.89	0.69–12.09	0.147

Abbreviations: UC, ulcerative colitis.

a Crude ORs of the conditional logistic regression were adjusted for age and gender.

b Adjusted ORs of the conditional logistic regression were adjusted for age, gender, and multiple comorbidities such as dyslipidemia, hypertension, ischemic heart disease, or chronic obstructive pulmonary disease.

### T-cell-specific Blimp-1 deficiency-mediated colitis is more severe in autoimmune diabetes-prone mice than in non-diabetes-prone mice

**Fig. 2:**
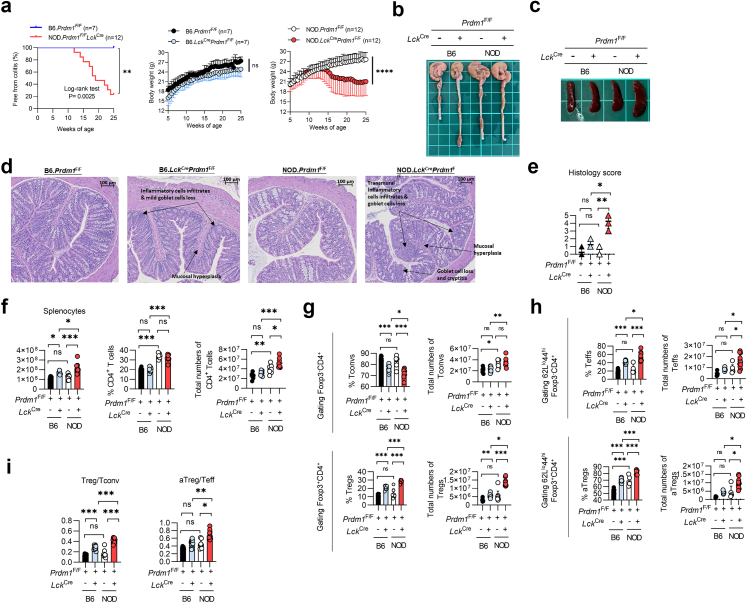
**Deletion of Blimp-1 in T cells augmentsthe ratio of Treg/Tconv cells and aTreg/Teff cells, especially in diabetes-prone mice. a.** Percentage of mice free from colitis and body weight changes in B6.*Lck*^*Cre*^*Prdm1*^F/F^ and NOD.*Lck*^*Cre*^*Prdm1*^F/F^ mice (hereafter referred to as Blimp-1 deficient) vs their littermate controls (*Prdm1*^F/F^), respectively (n = 7–14/group). **b and c.** Comparisons of colon length (b) and spleen size (c) between the groups indicated. **d and e.** Representative sections of the colon (d) and histological scoring (e) in the mice indicated. (Scale bar = 100 μm). **f.** Total splenocytes and CD4^+^ T-cell percentage and number in the groups indicated. **g–i.** Analysis of T cells: conventional T cell (Tconv), Treg, effector T cell (Teff), and activated Treg (CD62L^lo^CD44^hi^) frequencies (g), numbers (h), and population ratios (i). All analyses in (b–i) were performed in mice at 15–16 weeks of age, with n = 1 per group for (b–d), n = 4–8 per group for (e), and n = 6–13 per group for (f–i). Data represent the mean ± SEM of at least 3 independent experiments. Data were analyzed using the log-rank test for colitis-free percentage (a, left panel), a repeated measures ANOVA (a, middle and right panels), or 1-way ANOVA with Tukey's post hoc test (f, g upper left, g upper right, g lower left, h upper left, h lower left). For datasets deviating from normality, nonparametric alternatives [Mann–Whitney U test or Kruskal–Wallis test (e, g lower right, h upper right, h lower right)] were applied as appropriate. (∗*p* < 0.05, ∗∗*p* < 0.01, ∗∗∗*p* < 0.001; ns, not significant).

To explore further whether the autoimmune diabetes-prone genetic background affects the development of colitogenesis in our Blimp-1 deficiency-mediated colitis model, we characterised the disease kinetics and severity in autoimmune diabetes-prone NOD (NOD.*Lck*^*Cre*^*Prdm1*^*F/F*^) mice^17^ and diabetes-resistant C57BL/6 (B6.*Lck*^*Cre*^*Prdm1*^*F/F*^) mice.^13^ The loss of body weight and colitogenic onset started at 12–13 weeks of age in NOD.*Lck*^*Cre*^*Prdm1*^*F/F*^ mice, and 83% of these mice developed colitis at 25 weeks old, while B6.*Lck*^*Cre*^*Prdm1*^*F/F*^ mice remained disease-free (Fig. 2a), suggesting a much higher Blimp-1 deficiency-mediated colitogenic susceptibility in NOD mice than in C57BL/6 mice. NOD.*Lck*^*Cre*^*Prdm1*^*F/F*^ mice also exhibited greater colon thickening, splenomegaly, and more severe histological inflammation, including transmural infiltration and crypt damage compared to those in B6.*Lck*^*Cre*^*Prdm1*^*F/F*^ mice and their respective WT littermates (Fig. 2b–e). Taken together, our results from the nationwide epidemiological analysis and comparison of the disease phenotype in mice with Blimp-1 deficiency-mediated colitis between two genetic backgrounds indicate a higher prevalence of CD in people with T1D and an autoimmune diabetes-prone background-mediated promotion of colitogenesis.

### Lacking Blimp-1 in T cells augments the ratio of Tregs to Tconvs, especially in autoimmune diabetes-prone mice

To assess the impact of autoimmune diabetes-prone background-exacerbated colitogenesis on T cells, we analysed splenic T cell subpopulations in NOD.*Lck*^*Cre*^*Prdm1*^*F/F*^, B6.*Lck*^*Cre*^*Prdm1*^*F/F*^ mice and their WT littermates. The total numbers of splenocytes were similar between NOD and B6 mice but were significantly higher in NOD.*Lck*^Cre^*Prdm1*^*F/F*^ and B6.*Lck*^Cre^*Prdm1*^*F/F*^ mice than in their respective WT control. The difference in splenocyte number from the respective WT control was larger in NOD.*Lck*^Cre^*Prdm1*^*F/F*^ mice than in B6.*Lck*^Cre^*Prdm1*^*F/F*^ mice (Fig. 2f). The CD4^+^ T-cell percentage was significantly higher in diabetes-prone NOD mice than in non-diabetes-prone C57BL/6 mice (Fig. 2f). Although deletion of Blimp-1 did not augment CD4^+^ T-cell percentage in either group, the overall number of CD4^+^ T cells was much higher in Blimp-1 CKO NOD mice than in Blimp-1 CKO.C57BL/6 mice and WT NOD littermates (Fig. 2f).

Next, we measured the percentages and total cell numbers of Foxp3^–^CD4^+^ Tconvs and Foxp3^+^CD4^+^ Tregs. As percentages of Tconvs between NOD and B6 mice were comparable, percentages of Tconvs were significantly lower in Blimp-1 CKO mice from both genetic backgrounds than their control littermates. Meanwhile, percentages of Blimp-1 deficient Tconv in diabetes-prone mice was relatively lower than in non-diabetes-prone mice (Fig. 2g). However, the total numbers of Tconvs were higher in NOD mice than in B6 mice regardless of Blimp-1 deletion or not (Fig. 2g), as a result of higher total CD4^+^ T cell number in NOD mice than in B6 mice (Fig. 2f). Interestingly, Blimp-1 deletion augmented the percentage and total numbers of Tregs in both B6 and NOD mice and this increase was more significant in diabetes-prone mice than in non-diabetes-prone mice, whereas the Treg percentages and total numbers did not differ between WT NOD and C57BL/6 mice (Fig. 2g).

Similarly, Blimp-1 deletion significantly increased the percentages and total numbers of splenic CD44^hi^CD62L^lo^Foxp3^−^CD4^+^ effector T cells (Teffs) in both groups, in particular, the increase was greater in NOD.*Lck*^Cre^*Prdm1*^*F/F*^ mice than in B6.*Lck*^Cre^*Prdm1*^*F/F*^ mice (Fig. 2h). Interestingly, the percentages of CD44^hi^CD62L^lo^Foxp3^+^CD4^+^ Tregs (activated phenotype of Tregs, aTregs) was significantly higher in WT NOD mice than in C57BL/6 mice (Fig. 2h), suggesting that a compensatory increase in aTregs occurs during the autoimmune process in NOD mice. Consequently, the overall percentages and total numbers of aTregs in NOD.*Lck*^Cre^*Prdm1*^*F/F*^ mice were higher than in B6.*Lck*^Cre^*Prdm1*^*F/F*^ mice (Fig. 2h). Blimp-1 deletion significantly increased the ratio of Tregs/Tconvs in both strains, whereas the augmentation was significantly higher in NOD.*Lck*^Cre^*Prdm1*^*F/F*^ mice than in B6.*Lck*^Cre^*Prdm1*^*F/F*^ mice (Fig. 2i). Blimp-1 deletion also significantly increased the aTreg/Teff ratio in NOD mice but not in C57BL/6 mice compared with their WT NOD littermates and B6.*Lck*^Cre^*Prdm1*^*F/F*^ mice (Fig. 2i). Since *Lck*^*Cre*^ is expressed in all T cells, we further analysed CD4^+^ and CD8^+^ subsets. *Prdm1* deletion skewed the CD4:CD8 ratio toward a CD4^+^ predominance in both NOD and C57BL/6 backgrounds (Supplementary Fig. S1). NOD.*Lck*^Cre^*Prdm1*^*F/F*^ mice exhibited increased IFN-γ^+^ Th1 (*p* < 0.05, Kruskal–Wallis test) and IL-17A^+^ Th17 (*p* < 0.01, Kruskal–Wallis test) CD4^+^ subsets, with IFN-γ^+^ Th1 cells also elevated in B6.*Lck*^Cre^*Prdm1*^*F/F*^ vs WT (*p* < 0.05, Kruskal–Wallis test), but without significant differences between NOD.*Lck*^Cre^*Prdm1*^*F/F*^ and B6.*Lck*^Cre^*Prdm1*^*F/F*^ mice, aligning with our prior reports.^15^^,^^16^ These changes, together with Tregs dysfunction in NOD mice, may exacerbate colitogenesis in Blimp-1–deficient NOD mice.

Taken together, our results imply an autoimmune process-driven augmentation of Tregs and aTregs in diabetes-prone NOD mice. Interestingly, T-cell-specific Blimp-1 deficiency-mediated colitis was paradoxically more severe in mice with the NOD background than in C57BL/6 mice, despite the increased Treg/Tconv and aTreg/Teff ratios. As a result, we further addressed the suppressive function of Blimp-1-deficient Tregs in these mice.

### Deletion of Blimp-1 fragilizes the suppressive function of Tregs in autoimmune diabetes-prone mice

**Fig. 3:**
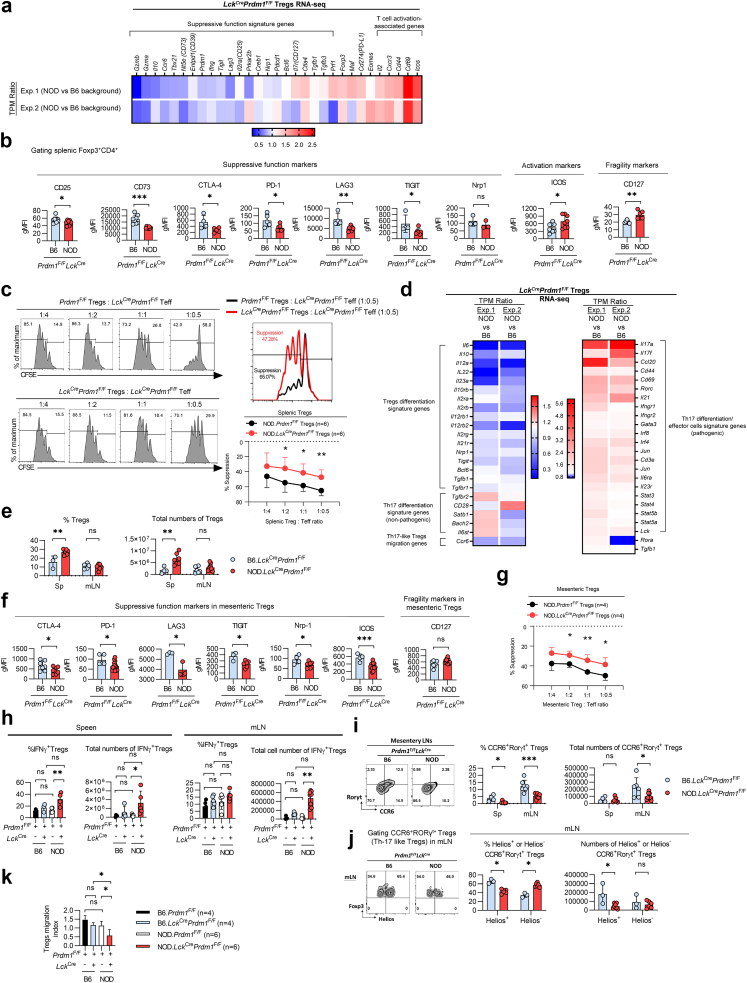
**T-cell-specific Blimp-1 deficiency significantly attenuates the expression of suppressive genes and function of Tregs and impairs migration of CCR6^+^Rorγt^+^Th17-like Tregs to mLNs in NOD compared with C57BL/6 mice. a.** Heatmap of RNA-seq analysis showing expression of Treg suppressive function genes in CD25^+^CD4^+^ splenic Tregs from 15-week-old NOD.*Lck*^*Cre*^*Prdm1*^F/F^ and B6.*Lck*^*Cre*^*Prdm1*^F/F^ mice (n = 2). **b.** Flow cytometry analysis of suppressive markers in NOD.*Lck*^*Cre*^*Prdm1*^F/F^ and B6.*Lck*^*Cre*^*Prdm1*^F/F^ Tregs (n = 6–9/group). **c.** In vitro suppression assay of splenic Tregs from *Prdm1*^F/F^ and NOD.*Lck*^*Cre*^*Prdm1*^F/F^ mice (n = 6/group). **d.** Heatmap comparing Th17-signature genes in NOD.*Lck*^*Cre*^*Prdm1*^F/F^ and B6.*Lck*^*Cre*^*Prdm1*^F/F^ Tregs (n = 2). **e.** Representative flow cytometry plots and quantification of Treg frequency and numbers in spleen and mLNs (n = 3–7/group). **f.** Flow cytometry analysis of suppressive markers in mLN Tregs (n = 4–9/group). **g.** Quantification of mesenteric Treg-suppression assay (n = 4). **h.** Representative flow cytometry plots and quantification of IFNγ^+^ Tregs in the spleen and mLNs (n = 4–8/group). (**i and j**) Representative plots and quantification of CCR6^+^RORγt^+^ Tregs (i, n = 4–8/group). and their Helios^+^ and Helios^–^ subsets (j, n = 6–8/group) in the tissues indicated. **k.** In vitro migration assay of splenic Tregs. The migration index represents Tregs migrating toward CCL20 compared with the medium control (n = 4–6/group). Data represent the mean ± SEM of at least 3 independent experiments. The data were analysed using a 2-tailed Student's *t*-test (b: CD25, CD73, CTLA-4, LAG3, ICOS, CD127, c, e-f, i: CTLA-4, LAG3, TIGIT, Nrp-1, ICOS, CD127 and j) or multiple Student's *t*-tests (g) or 1-way ANOVA with Tukey's post hoc test (h: % of IFNγ+ Tregs in spleen and mLN, total numbers of IFNγ+ Tregs in spleen and k). For datasets deviating from normality, nonparametric alternatives [Mann–Whitney U test (b: Nrp1, PD-1 and TIGIT; c: PD-1; i, j) or Kruskal–Wallis test (h: total numbers of IFNγ+ Tregs in spleen)] were applied as appropriate. (∗*p* < 0.05, ∗∗*p* < 0.01, ∗∗∗*p* < 0.001; ns, not significant).

Given the critical role of Blimp-1 for controlling the suppressive function of Tregs,^13^^,^^31^ we next compared the potential impact of Blimp-1 deficiency-mediated functional impairment of Tregs between NOD and C57BL/6 mice by performing bulk RNA-seq of splenic Tregs from NOD.*Lck*^Cre^*Prdm1*^*F/F*^ and B6.*Lck*^Cre^*Prdm1*^*F/F*^ mice. Multiple suppressive function genes, including cytolysis-inducing genes (*Gzmb*, *Gzma*), inhibitory cytokines (*Il10*), metabolic disruption mediators (*Nt5e*, *Entpd1*, *Il2ra*), and DC maturation inhibitors (*Lag3*, *Tigit*, *Nrp1*), were significantly downregulated in *Lck*^Cre^*Prdm1*^*F/F*^ Tregs with the NOD background compared with C57BL/6 mice (Fig. 3a), consistent with flow cytometry data showing that the lower expression levels of CD25, CD73, CTLA-4, PD-1, LAG3, and TIGIT in NOD.*Lck*^*Cre*^*Prdm1*^*F/F*^ Tregs (Fig. 3b). Expression level of CD127, the Treg fragility marker inversely correlated with Treg suppressive function,^32^ was significantly increased in NOD.*Lck*^*Cre*^*Prdm1*^*F/F*^ Tregs than in B6.*Lck*^*Cre*^*Prdm1*^*F/F*^ Tregs (Fig. 3b). T-cell activation-associated gene transcripts (*Il2*, *Cxcr3*, *Cd44*, *Cd69*, and *Icos*) were significantly upregulated in Blimp-1-deficient Tregs from NOD mice compared with those from C57BL/6 mice (Fig. 3a), consistent with the flow cytometry data (Fig. 3b) and suggesting stronger TCR signaling and subsequent activation potential in NOD.*Lck*^*Cre*^*Prdm1*^*F/F*^ Tregs than in B6.*Lck*^*Cre*^*Prdm1*^*F/F*^ Tregs. Our functional assay of NOD Tregs revealed that Blimp-1 deficiency induced a defective suppression of the proliferation of Teffs (CKO vs WT Tregs: 47.28% vs 65.07%) at a 1:0.5 ratio of splenic Tregs/Teffs (Fig. 3c). In contrast, Blimp-1–deficient C57BL/6 Tregs suppressed Teffs to levels indistinguishable from WT C57BL/6 Tregs, indicating that the suppression defect in NOD Tregs is largely driven by strain-specific background rather than Blimp-1 loss alone (Supplementary Fig. S2). Taken together, these data indicate that loss of Blimp-1 in Tregs fragilizes their immunosuppressive function through the upregulation of TCR signaling and activation of genes in autoimmune diabetes-prone NOD mice.

### Lack of Blimp-1 in Tregs promotes stronger Th17-like signature gene expression and reduces migration of Th17-like Tregs to the mLNs in autoimmune diabetes-prone mice compared with non-diabetes-prone mice

Since Blimp-1 is required to repress Th17-associated cytokines in CCR6^+^RORγt^+^Th17-like Tregs,^22^^,^^31^ we investigate the impact of Blimp-1 loss on transcriptomic landscape of Th17-like Tregs in C57BL/6 and NOD mice. Interestingly, a panel of maturation and differentiation-associated genes in Tregs, such as *Il6*, *Il10*, *Il10rb*, *Il22*, *Il23a*, *Nrp1*, and *Tgfb1*,^33^, ^34^, ^35^, ^36^ expressed as the ratio of the TPM of gene expression, was significantly downregulated in NOD.*Lck*^Cre^*Prdm1*^*F/F*^ mice compared with B6.*Lck*^Cre^*Prdm1*^*F/F*^ mice^34^^,^^36^ (Fig. 3d, left), while pathogenic Th17 differentiation/effector signature genes such as *Il17a*, *Il17f*, *Il21*, *Cd44*, *Ccl20*, *Rorc*, *Gata3*, *Irf4*, *Irf8*, *Il6ra*, *Il23r*, *Stat3*, *Stat4*, *Stat5a*, and *Stat5b*^36^ were significantly upregulated (Fig. 3d, right). Notably, the Th17-like Treg migration signature gene (Ccr6) was significantly downregulated in Blimp-1-deficient Tregs in NOD mice compared with C57BL/6 mice (Fig. 3d).

To explore further the impact of Blimp-1 deficiency on the migratory function of Tregs, we compared Tregs population in the spleen and mLNs between NOD and C57BL/6 mice. The percentages and total numbers of splenic Tregs were much higher in NOD*.Lck*^*Cre*^*Prdm1*^*F/F*^ than in B6.*Lck*^*Cre*^*Prdm1*^*F/F*^ mice, whereas those of mesenteric Tregs were similar in both groups (Fig. 3e), suggesting a lower capability of the Treg gut-homing function in NOD.*Lck*^*Cre*^*Prdm1*^*F/F*^ than in B6.*Lck*^*Cre*^*Prdm1*^*F/F*^ mice.

The expression levels of representative markers of Treg suppressive function, such as CTLA-4, PD-1, LAG-3, TIGIT, Nrp-1, and ICOS, were also significantly lower in mesenteric Tregs in NOD.*Lck*^*Cre*^*Prdm1*^*F/F*^ than in B6.*Lck*^*Cre*^*Prdm1*^*F/F*^ mice (Fig. 3f). Moreover, Treg-suppression assay revealed that mesenteric NOD.*Lck*^*Cre*^*Prdm1*^*F/F*^ Tregs exhibited impaired ability to suppress mesenteric Teff proliferation compared to WT controls (Fig. 3g).

IFNγ^+^ Tregs have been reported to promote antitumor responses by loss of their suppressive function.^37^ The total numbers of splenic and mesenteric IFNγ^+^ Tregs were significantly higher in NOD.*Lck*^*Cre*^*Prdm1*^*F/F*^ than in their NOD WT littermate controls, but the percentages and total numbers of IFNγ^+^ Tregs from the spleens and mLNs were comparable between B6.*Lck*^*Cre*^*Prdm1*^*F/F*^ mice and controls (Fig. 3h), supporting the idea of a more fragile phenotype of Blimp-1-deficient Tregs with lower suppressive activity for controlling intestinal inflammation in mice from the autoimmune diabetes-prone background than in non-diabetes-prone mice.

Next, we investigate the impact of Blimp-1 deficiency on the migration of gut-homing Th17-like (CCR6^+^RORγt^+^Foxp3^+^) Tregs and found significantly lower percentages and total numbers of Blimp-1-deficient Th17-like Tregs from mLNs of NOD.*Lck*^*Cre*^*Prdm1*^*F/F*^ than of B6.*Lck*^*Cre*^*Prdm1*^*F/F*^ mice (Fig. 3i). These results suggest that Blimp-1 deficiency impedes the migratory function of gut-homing Th17-like Tregs to mLNs, resulting in a lower ability to suppress colitis in NOD mice than in C57BL/6 mice.

Given that Tregs can be grouped into natural Tregs (Helios^+^nTregs) and tissue-resident inducible Tregs (Helios^–^ iTregs) based on their different origins,^38^ we explored whether reduced mesenteric Th17-like Tregs in Blimp-1-deficient NOD mice results from the impairment of splenic nTreg migration or lower inducibility of tissue-resident iTregs or both. We analysed mesenteric Tregs by gating Helios^+^CCR6^+^RORgt^+^Foxp3^+^ Tregs as thymic-derived gut-homing nTregs and Helios^−^CCR6^+^RORγt^+^Foxp3^+^ Tregs as peripherally generated iTregs. Although the percentages of Helios^–^ peripherally induced Th17-like Tregs was higher in NOD.*Lck*^*Cre*^*Prdm1*^*F/F*^ than in B6.*Lck*^*Cre*^*Prdm1*^*F/F*^ mice, the overall numbers of these iTregs in mLNs was quite low and indistinguishable between the 2 genetic backgrounds (Fig. 3j). The percentages and total numbers of Helios^+^ migratory natural gut-homing Th17-like Tregs in mLNs were much lower in NOD*.Lck*^*Cre*^*Prdm1*^*F/F*^ mice than in B6.*Lck*^*Cre*^*Prdm1*^*F/F*^ mice (Fig. 3j), supporting that the impairment of splenic Blimp-1-deficient nTreg migration toward colonic inflammatory sites in diabetes-prone mice compared with non-diabetes-prone mice. Furthermore, the migration index was significantly lower in splenic Tregs from NOD.*Lck*^*Cre*^*Prdm1*^*F/F*^ mice than from their littermate controls and B6.*Lck*^*Cre*^*Prdm1*^*F/F*^ mice by using in vitro migration assay (Fig. 3k). This finding demonstrates that deletion of Blimp-1 significantly impedes the migratory ability of gut-homing Tregs in a more severe manner in autoimmune diabetes-prone mice than in non-diabetes-prone mice.

### Blimp-1 deficiency enhances the TCR signaling strength of Tregs in autoimmune diabetes-prone mice

**Fig. 4:**
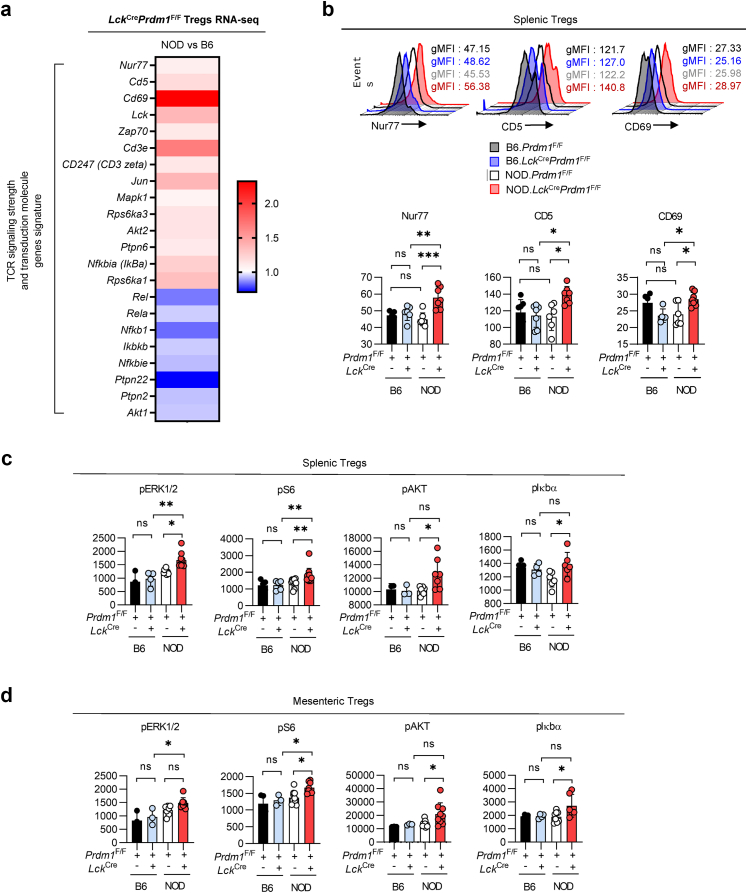
**T-cell-specific Blimp-1 deficiency increases the TCR signaling strength of Tregs in NOD mice compared with WT littermates and T-cell-specific Blimp-1-deficient B6 mice. a.** RNA-seq analysis of Tregs from NOD.*Lck*^*Cre*^*Prdm1*^F/F^ and B6.*Lck*^*Cre*^*Prdm1*^F/F^ mice (n = 1). **b.** Flow cytometry analysis of Nur77, CD5, and CD69 expression in splenic Tregs (n = 6–8/group). **c and d.** TCR downstream signaling in Tregs and Tconvs from the spleen (c, n = 3–13/group) and mLNs (d, n = 3–10/group). All analyses were performed in mice aged 14–16 weeks. Data represent the mean ± SEM of at least 3 independent experiments. The data were analyzed using 1-way ANOVA with Tukey's post hoc test (b, c: pAKT, pIκbα and d). For datasets deviating from normality, nonparametric alternatives [Kruskal–Wallis test (c: pERK1/2, pS6)] were applied as appropriate. (∗*p* < 0.05, ∗∗*p* < 0.01, ∗∗∗*p* < 0.001; ns, not significant).

Since TCR signaling is required for Treg suppressive function^24^ and a distinct population of highly self-reactive Tregs (Nur77^hi^CD5^hi^) with increased TCR signaling losses its suppressive capacity to protect recipients from colon inflammation in adoptive transfer models,^25^ we examine whether the suppressive discrepancy between Blimp-1-deficient NOD Tregs and B6 Tregs correlates with their TCR signaling strength by RNA-seq and flow cytometry analysis. Interestingly, we found a significant upregulation of a panel of genes relating to TCR signaling strength (Nur77 and CD5), and the T-cell activation gene CD69 in NOD.*Lck*^*Cre*^*Prdm1*^*F/F*^ mice compared with B6.*Lck*^*Cre*^*Prdm1*^*F/F*^ mice (Fig. 4a), suggesting an inverse correlation between TCR signaling strength and its suppressive capability in NOD Tregs. Consistent with the abovementioned findings, a series of pivotal TCR signal transduction molecule gene (*CD3e*, *CD3z*, *Lck*, *Zap70*, *Jun*, *Mapk1*, *Akt2*, *Nfkbia*, and *Rps6ka1*^39^, ^40^, ^41^) were upregulated in Tregs of NOD.*Lck*^*Cre*^*Prdm1*^*F/F*^ mice compared with B6.*Lck*^*Cre*^*Prdm1*^*F/F*^ mice. Additionally, the expression of NF-κB pathway-related genes (*Rel*, *Rela*, *Ikbkb*, *Nfkbie*, and *Nfkb1*) were lower in Blimp-1-deficient Tregs in NOD than in C57BL/6 mice (Fig. 4a), consistent with previous reports that NF-κB c-Rel ablation in Tregs impaired their suppressive function and markedly reduced melanoma growth.^42^^,^^43^

Strikingly, our data revealed that *Ptpn22*, which encodes a phosphatase that acts as an attenuator of TCR signaling strength,^44^, ^45^, ^46^ was significantly downregulated in NOD.*Lck*^*Cre*^*Prdm1*^*F/F*^ compared with B6.*Lck*^*Cre*^*Prdm1*^*F/F*^ Tregs (Fig. 4a). This finding suggests that NOD.*Lck*^*Cre*^*Prdm1*^*F/F*^ Tregs sustain a higher TCR signaling strength than B6.*Lck*^*Cre*^*Prdm1*^*F/F*^ Tregs. Data from flow cytometry analyses further support the idea that Blimp-1 deficiency-mediated augmentation of CD5, Nur77, and CD69 is specific to the NOD background because the expression of these molecules was significantly higher in NOD.*Lck*^*Cre*^*Prdm1*^*F/F*^ than in B6.*Lck*^*Cre*^*Prdm1*^*F/F*^ Tregs (Fig. 4b). We subsequently elucidated phosphorylation levels of downstream TCR signaling molecules in splenic and mesenteric (Fig. 4c and d) Tregs from NOD.*Lck*^*Cre*^*Prdm1*^*F/F*^ and B6.*Lck*^*Cre*^*Prdm1*^*F/F*^ mice. We observed a NOD strain-specific augmentation of phosphorylation levels of ERK1/2, S6, AKT, and Iκbα in Blimp-1-deficient Tregs. Collectively, our results suggest that Blimp-1 deficiency significantly upregulates TCR signaling strength, downregulates *Ptpn22* expression levels, and increases TCR signaling molecules in Tregs, particularly in autoimmune diabetes-prone mice.

### Augmentation of Pep restores the suppressive function of Blimp-1-deficient Tregs by attenuating its TCR signaling strength and subsequently ameliorates the Blimp-1 deficiency-mediated colitis in diabetes-prone mice

**Fig. 5:**
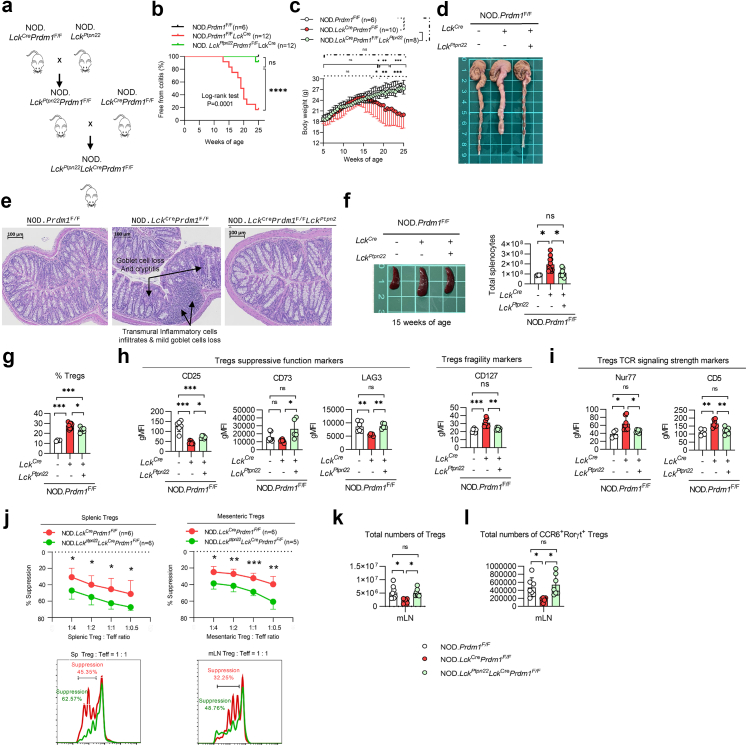
**Lck-driven *Ptpn22* overexpression ameliorates T-cell-specific Blimp-1 deficiency-mediated colitis in NOD mice. a.** Generation scheme of NOD.*Lck*^Cre^*Prdm1*^F/F^*Lck*^*Ptpn22*^ mice. **b.** Percentage of colitis-free mice in NOD.*Lck*^Cre^*Prdm1*^F/F^ and NOD.*Lck*^Cre^*Prdm1*^F/F^*Lck*^*Ptpn22*^ mice (n = 6–12/group). **c.** Body weight changes in the groups indicated (n = 6–10/group). **d–f.** Representative images of colon length (d), colon sections (scale bar = 100 μm) (e), and spleen size (f, n = 4–11/group) from NOD.*Prdm1*^F/F^, NOD.*Lck*^Cre^*Prdm1*^F/F^, and NOD.*Lck*^Cre^*Prdm1*^F/F^*Lck*^*Ptpn22*^ mice at 15 weeks of age. **g–i.** Analysis of splenic Treg frequency (g, n = 4–9/group), suppressive function markers (h, n = 5–8/group), and markers of TCR signaling strength (i, n = 4–7/group) in the groups indicated. **j.** In vitro Treg-suppression assay comparing splenic Tregs (n = 6/group) and mesenteric Tregs (n = 5–6/group) from NOD.*Prdm1*^F/F^, NOD.*Lck*^Cre^*Prdm1*^F/F^, and NOD.*Lck*^Cre^*Prdm1*^F/F^*Lck*^*Ptpn22*^ mice. **k and l.** Quantification of total Tregs (k, n = 6–8/group) and CCR6^+^RORγt^+^ Tregs (l, n = 6–7/group) in mLNs. All analyses in g-l were performed at 14–16 weeks of age. Data represent the mean ± SEM of at least 3 independent experiments; significance was determined using the log-rank test (b), 2-way ANOVA with Tukey's post hoc test (c), 1-way ANOVA with Tukey's post hoc test (f, g, h: CD25, LAG3, CD127, i, k, l), or a 2-tailed Student's *t*-test (j). For datasets deviating from normality, nonparametric alternatives [Kruskal–Wallis test (h: CD73)] were applied as appropriate. (∗*p* < 0.05, ∗∗*p* < 0.01, ∗∗∗*p* < 0.001; ns, not significant).

Given our findings that a negative correlation between TCR signaling strength and the suppressive function and that a significant downregulation of Pep in NOD.*Lck*^*Cre*^*Prdm1*^*F/F*^ Tregs compared with B6.*Lck*^*Cre*^*Prdm1*^*F/F*^ Tregs, we hypothesised that downregulation of TCR signaling strength by overexpression of Pep would restore the suppressive function of NOD.*Lck*^*Cre*^*Prdm1*^*F/F*^ Tregs. To test this hypothesis, we crossed T cell-specific Pep transgenic mice (NOD.*Lck*^*Ptpn22*^) with NOD.*Lck*^*Cre*^*Prdm1*^*F/F*^ mice to generate NOD.*Lck*^*Ptpn22*^*Lck*^*Cre*^*Prdm1*^*F/F*^ mice (Fig. 5A). Compared to NOD.*Lck*^*Cre*^*Prdm1*^*F/F*^ mice, NOD.*Lck*^*Ptpn22*^*Lck*^*Cre*^*Prdm1*^*F/F*^ mice showed delayed colitogenic onset by about 13 weeks, significantly higher colitis-free percentages (91.6% vs 16.6% at 25 weeks), and no significant body weight loss (Fig. 5b and c). Additionally, the length of the colon was longer without signs of inflammation (Fig. 5d) and colonic sections at 15 weeks old showed reduced inflammatory cell infiltration with more preserved goblet cells in NOD.*Lck*^*Ptpn22*^*Lck*^*Cre*^*Prdm1*^*F/F*^ mice compared with NOD.*Lck*^*Cre*^*Prdm1*^*F/F*^ mice (Fig. 5e), suggesting that Pep overexpression rendered significant protection from Blimp-1 deficiency-mediated colitis. The spleen size and total numbers of splenocytes in NOD.*Lck*^*Ptpn22*^*Lck*^*Cre*^*Prdm1*^*F/F*^ mice were similar to those in the NOD controls owing to Pep overexpression (Fig. 5f). Interestingly, splenic NOD*.Lck*^*Ptpn22*^*Lck*^*Cre*^*Prdm1*^*F/F*^ Tregs population was decreased (Fig. 5g) with restored suppressive markers (CD25, CD73 and LAG3), reduced fragility marker CD127 (Fig. 5h), and reduced TCR signaling strength markers (Nur77 and CD5) compared with controls (Fig. 5i).

To examine further whether Pep overexpression restores impaired Treg function in NOD*.Lck*^*Cre*^*Prdm1*^*F/F*^ mice, we conducted a Treg-suppression assay. We found a significant regain of suppressive function of splenic and mesenteric Tregs from NOD*.Lck*^*Ptpn22*^*Lck*^*Cre*^*Prdm1*^*F/F*^ compared with NOD*.Lck*^*Cre*^*Prdm1*^*F/F*^ mice (Fig. 5j). In mLNs, the total numbers of Tregs and CCR6^+^RORγt^+^ Th17-like Tregs in NOD*.Lck*^*Ptpn22*^*Lck*^*Cre*^*Prdm1*^*F/F*^ mice were restored to the level of the control NOD*.Prdm1*^*F/F*^ mice (Fig. 5k and l). Taken together, our results suggest that augmentation of Pep in Blimp-1-deficient NOD Tregs significantly rescues their suppressive function and increases the population of Th17-like Tregs, which subsequently ameliorates colitogenesis and disease severity.

To determine whether the mechanistic signature observed in our murine model is recapitulated in human T1D, we re-analyzed whole-blood transcriptomic data from 39 T1D patients and 43 healthy controls (GSE123658; Supplementary Fig. S3). T1D samples showed reduced *PRDM1* and core suppressive markers, such as *ENTPD1/CD39, LAG3, NRP1, TBX21*, but increased activation markers, including *CD44, CD69, ICOS, CXCR3*, pro-inflammatory Th17-associated transcripts, such as *GATA3, IL17B, IL23R, RORA* and TCR signaling genes, including *LCK, NR4A1/Nur77, ZAP70*, with concurrent *IL6* downregulation, aligning with the TCR hyperactivation model we proposed. Notably, *PTPN22* (PEP) was modestly increased, suggesting a residual compensatory response aimed at dampening excessive TCR signaling and sustaining Treg stability. Such compensation was only observed in human T1D because *PRDM1* expression was reduced but not completely lost, enabling *PTPN22* to partially counterbalance heightened TCR signaling. In contrast, in our murine NOD model, *Blimp-1 (Prdm1)* is completely ablated in Tregs, eliminating this regulatory axis and precluding such compensation. Despite originating from whole blood rather than purified Tregs, these convergent transcriptional patterns reinforce the translational relevance of the Blimp-1/PEP/TCR signaling axis in T1D.

### Transgenic Pep-mediated protection in NOD.*Lck*^*Ptpn22*^*Lck*^*Cre*^*Prdm1*^*F/F*^ mice is Treg-dependent

**Fig. 6:**
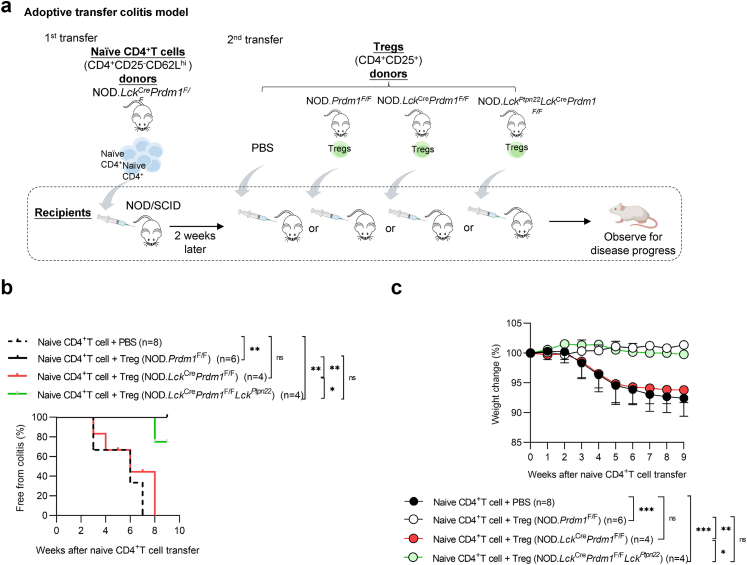
**T-cell-specific Pep overexpression in Blimp-1-deficient Tregs protects NOD/SCID recipients from colitogenesis in an adoptive transfer model. a.** Adoptive transfer of CD4^+^CD25^–^CD62L^hi^ naïve T cells from NOD.*Lck*^Cre^*Prdm1*^F/F^ mice into NOD/SCID recipients, followed by CD4^+^CD25^+^ Tregs from NOD.*Prdm1*^F/F^, NOD.*Lck*^Cre^*Prdm1*^F/F^, NOD.*Lck*^*Ptpn22*^*Lck*^Cre^*Prdm1*^F/F^, or PBS-injected mice after 2 weeks (n = 4–8 mice per group). **b.** Cumulative incidence curve of diarrhea in NOD/SCID recipients after adoptive transfer. **c.** Body weight changes in NOD/SCID recipients following transfer of CD4^+^CD25^+^ Tregs from NOD.*Prdm1*^F/F^, NOD.*Lck*^Cre^*Prdm1*^F/F^, NOD.*Lck*^*Ptpn2*^*Lck*^Cre^*Prdm1*^F/F^, or PBS-injected mice (n = 4–8 mice per group). Data represent the mean ± SEM of at least 3 independent experiments. Statistical significance was determined using the log-rank test for diarrhea incidence (b). For body weight changes (c), statistical analysis was performed using the Kruskal–Wallis test as a nonparametric alternative due to deviation from normality. (∗*p* < 0.05, ∗∗*p* < 0.01, ∗∗∗*p* < 0.001).

**Fig. 7:**
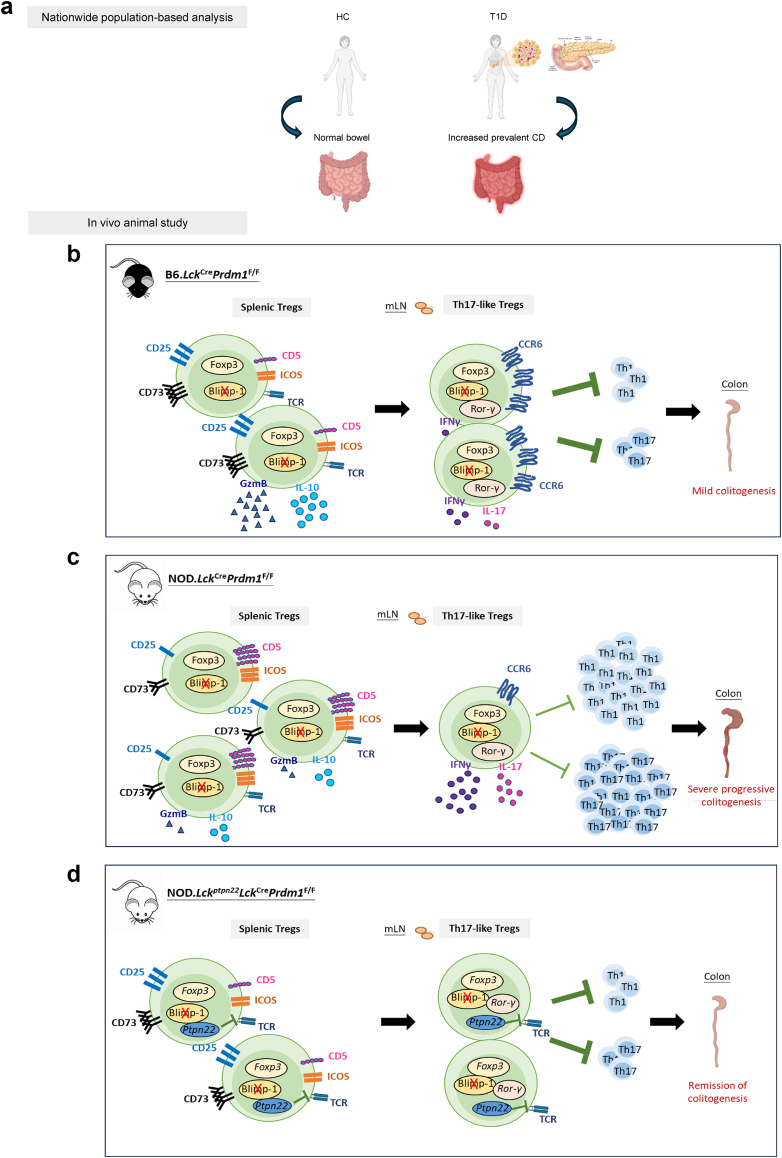
**Integration of epidemiological data with molecular mechanisms of Blimp-1 in Treg-mediated immune regulation.** Schematic diagrams illustrate the integration of the nationwide population-based epidemiological data. **a.** and data from the experimental models. **b and c.** The diagram reveals a newly described link between T1D and IBD that highlights the critical role of Blimp-1 in Treg regulation of TCR-modulated suppressive function and the migration of gut-homing Th17-like Tregs toward mLNs to counterbalance Th17-mediated colitis in non-diabetes-prone mice (b) and diabetes-prone mice (c). **d.** The diagram also demonstrates how transgenic Pep-mediated TCR inhibition restores the defective function of gut-homing Blimp-1-deficient Tregs in NOD mice.

To dissect further whether the colitogenic protection in NOD.*Lck*^*Ptpn22*^*Lck*^*Cre*^*Prdm1*^*F/F*^ mice is mediated through a Treg-associated manner, we performed a 2-step adoptive transfer colitis and Treg-suppression model. At two weeks after the transfer of naïve Blimp-1-deficient CD4^+^ T cells, we intraperitoneally injected Tregs from indicated donors (Fig. 6a). Recipients receiving PBS or NOD.*Lck*^*Cre*^*Prdm1*^*F/F*^ Tregs quickly developed severe colitis (∼40% by week three) with progressive weight loss reaching its lowest point around 6–8 weeks post-transfer (Fig. 6b and c). By contrast, the body weights of NOD*.Lck*^*Ptpn22*^*Lck*^*Cre*^*Prdm1*^*F/F*^ Treg-transferred recipients decreased by only a small amount (Fig. 6c). In summary, our in vivo data provides solid evidence that Pep overexpression can rescue the impaired suppressive function of Blimp-1-deficient Tregs (Fig. 7) and suggests the potential of Pep-mediated TCR signaling inhibition for controlling autoimmune diseases.

## Discussion

The devastating acute and chronic complications and their effects on quality of life in people with autoimmune diseases are increasing worldwide.^47^^,^^48^ In this study, we found evidence of an essential role of Blimp-1 in sustaining Treg suppressive function and a critical role of Pep-mediated TCR signaling attenuation in restoring the impaired function of Blimp-1-deficient Tregs in autoimmune diabetes-prone mice.

Blimp-1 is vital for Treg lineage stability, and its loss promotes Foxp3 downregulation and conversion of Tregs into proinflammatory ex-Tregs.^31^ Our findings reveal that Blimp-1-deficient Tregs from diabetes-prone NOD mice exhibited a more fragile phenotype involving a reduced capacity to suppress Teff proliferation and migration, particularly in gut-homing Th17-like Tregs compared with Tregs from non-diabetes-prone C57BL/6 mice. Blimp-1-deficient Tregs showed increased IFN-γ and IL-17 production, which reinforced their pathogenic phenotype in autoimmune diabetes-prone NOD mice. This aligns with previous findings that Blimp-1 represses the inflammatory gene landscape by directly suppressing *Il2*, *Il17*, and *Bcl6*,^31^^,^^49^^,^^50^ which help maintain Treg immunosuppressive functions.

Previous reports have shown that sustained TCR signaling drives T cells toward an exhausted phenotype, which ultimately results in a gradual loss of Treg effector function and impaired suppression of Teff proliferation in both tumor and autoimmune disease models.^51^^,^^52^ In autoimmune diabetic mice, it is likely that excessive TCR signaling contributes to a decreased Treg suppressive capacity. Previous report demonstrated that NF-κB c-Rel ablation impairs Treg function and delayed melanoma growth.^43^ Consistently, our findings show significant downregulation of NF-κB pathway genes, such as *Rel*, *Rela*, *Ikbkb*, *Nfkbie*, and *Nfkb1*, in Blimp-1-deficient NOD Tregs possessing impaired suppressive function relative to those from C57BL/6 mice. Increased TCR signaling resulting from *PTPN22* deficiency promotes autoreactive T-cell development and β-cell destruction in the pancreas,^53^ whereas autoimmune-associated *PTPN22* variants impair Treg suppression.^54^^,^^55^ Although the impact of the R620W variant on TCR signaling remains debatable, some reports have suggested a gain-of-function^56^ and others a loss-of-function^54^ effect; these different findings underscore the complexity of the role of *PTPN22*.

Treg's suppressive capacity is intricately linked to downstream TCR signaling, including the MAPK and mTOR pathways.^57^^,^^58^ Inhibition of ERK phosphorylation enhances Treg function, as evidenced by the UO126-induced *Foxp3* expression associated with transient ERK inhibition,^59^ suggesting that inhibition of the ERK pathway may promote Treg suppressive function through epigenetic mechanisms. Similarly, the inhibition of mitochondrial enzyme increased mTOR signaling in Tregs to decrease their suppressive capacity.^60^ In our present study, Blimp-1-deficient NOD Tregs exhibit elevated mTOR signaling to impair their suppressive function.

Our human transcriptomic analysis (GSE123658; Supplemental Fig. S3), recapitulating the Blimp-1/PEP/TCR signaling axis in T1D pathophysiology, highlighting its translational relevance and therapeutic potential. However, our transcriptomic analysis using human samples was limited by the sample source; further confirmatory phenotyping could enhance the validity of our findings regarding the use of patient-derived Tregs to dissect cell-intrinsic mechanisms.

These results provide new insights into the molecular mechanisms of gut-homing Treg dysfunction in T1D and highlight Pep as a promising therapeutic target. A limitation of this study was that race and ethnicity data were not available in the NHIRD; however, the database covers over 99% of Taiwan's population, ensuring representativeness of the study cohort and providing valuable evidence to inform strategies that promote health equity and preventive care. Future studies should assess the translational potential of modulating PEP in human Tregs to improve Treg-based immunotherapies and help prevent autoimmune progression in people with T1D.

## Contributors

HKS was involved in study conceptualisation and design, as well as in the development of the transgenic NOD mouse methodology. YWT, YWL, CYH, SHF, MWC, and JLD were involved in the investigation. YWL, CYH, SHF, MWC, and JLD were responsible for data curation. YWT, CCS, and CTC conducted the formal analyses. HKS provided supervision. YWT and HKS drafted the original manuscript. HKS was involved in the review and editing of the manuscript. YWT and HKS had full access to and verified the underlying data. Dr. HKS was responsible for the decision to submit the manuscript. All authors read and approved the final version of the manuscript.

## Data sharing statement

This study is based on data from Taiwan's National Health Insurance Research Database, provided by the National Health Insurance Administration, Ministry of Health and Welfare, and maintained electronically by the National Health Research Institute and the National Health Insurance Administration. Any use of the raw data requires a license agreement with the National Health Insurance Administration in Taiwan.

## Declaration of interests

The authors declare no conflict of interest.
