# Supplementary material for: Blimp-1 benefits gut-homing regulatory T cells by maintaining migration/suppressive function in autoimmune diabetes-prone mice
Source: eBioMedicine. 2025 Nov 5;121:106002. doi: 10.1016/j.ebiom.2025.106002 (PMC12636379; doi:10.1016/j.ebiom.2025.106002)
Supplement: Supplementary Data [file mmc1.pptx]

## Slide 1
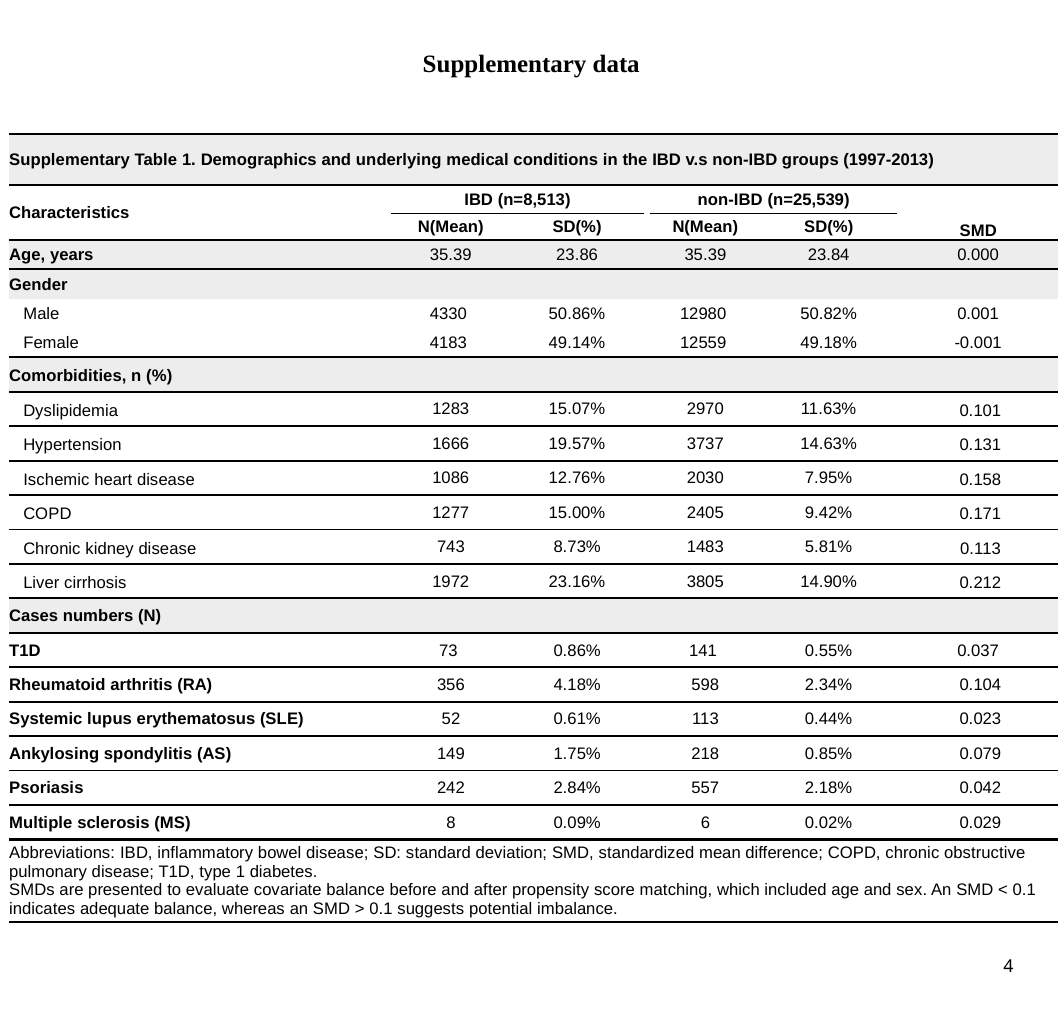

Supplementary data
| Supplementary Table 1. Demographics and underlying medical conditions in the IBD v.s non-IBD groups (1997-2013) | | | | | | | |
| --- | --- | --- | --- | --- | --- | --- | --- |
| Characteristics | IBD (n=8,513) | | | non-IBD (n=25,539) | | | SMD |
| | N(Mean) | SD(%) | | N(Mean) | SD(%) | | |
| Age, years | 35.39 | 23.86 | | 35.39 | 23.84 | | 0.000 |
| Gender | | | | | | | |
| Male | 4330 | 50.86% | | 12980 | 50.82% | | 0.001 |
| Female | 4183 | 49.14% | | 12559 | 49.18% | | -0.001 |
| Comorbidities, n (%) | | | | | | | |
| Dyslipidemia | 1283 | 15.07% | | 2970 | 11.63% | | 0.101 |
| Hypertension | 1666 | 19.57% | | 3737 | 14.63% | | 0.131 |
| Ischemic heart disease | 1086 | 12.76% | | 2030 | 7.95% | | 0.158 |
| COPD | 1277 | 15.00% | | 2405 | 9.42% | | 0.171 |
| Chronic kidney disease | 743 | 8.73% | | 1483 | 5.81% | | 0.113 |
| Liver cirrhosis | 1972 | 23.16% | | 3805 | 14.90% | | 0.212 |
| Cases numbers (N) | | | | | | | |
| T1D | 73 | 0.86% | | 141 | 0.55% | | 0.037 |
| Rheumatoid arthritis (RA) | 356 | 4.18% | | 598 | 2.34% | | 0.104 |
| Systemic lupus erythematosus (SLE) | 52 | 0.61% | | 113 | 0.44% | | 0.023 |
| Ankylosing spondylitis (AS) | 149 | 1.75% | | 218 | 0.85% | | 0.079 |
| Psoriasis | 242 | 2.84% | | 557 | 2.18% | | 0.042 |
| Multiple sclerosis (MS) | 8 | 0.09% | | 6 | 0.02% | | 0.029 |
| Abbreviations: IBD, inflammatory bowel disease; SD: standard deviation; SMD, standardized mean difference; COPD, chronic obstructive pulmonary disease; T1D, type 1 diabetes. SMDs are presented to evaluate covariate balance before and after propensity score matching, which included age and sex. An SMD < 0.1 indicates adequate balance, whereas an SMD > 0.1 suggests potential imbalance. | | | | | | | |
4

## Slide 2
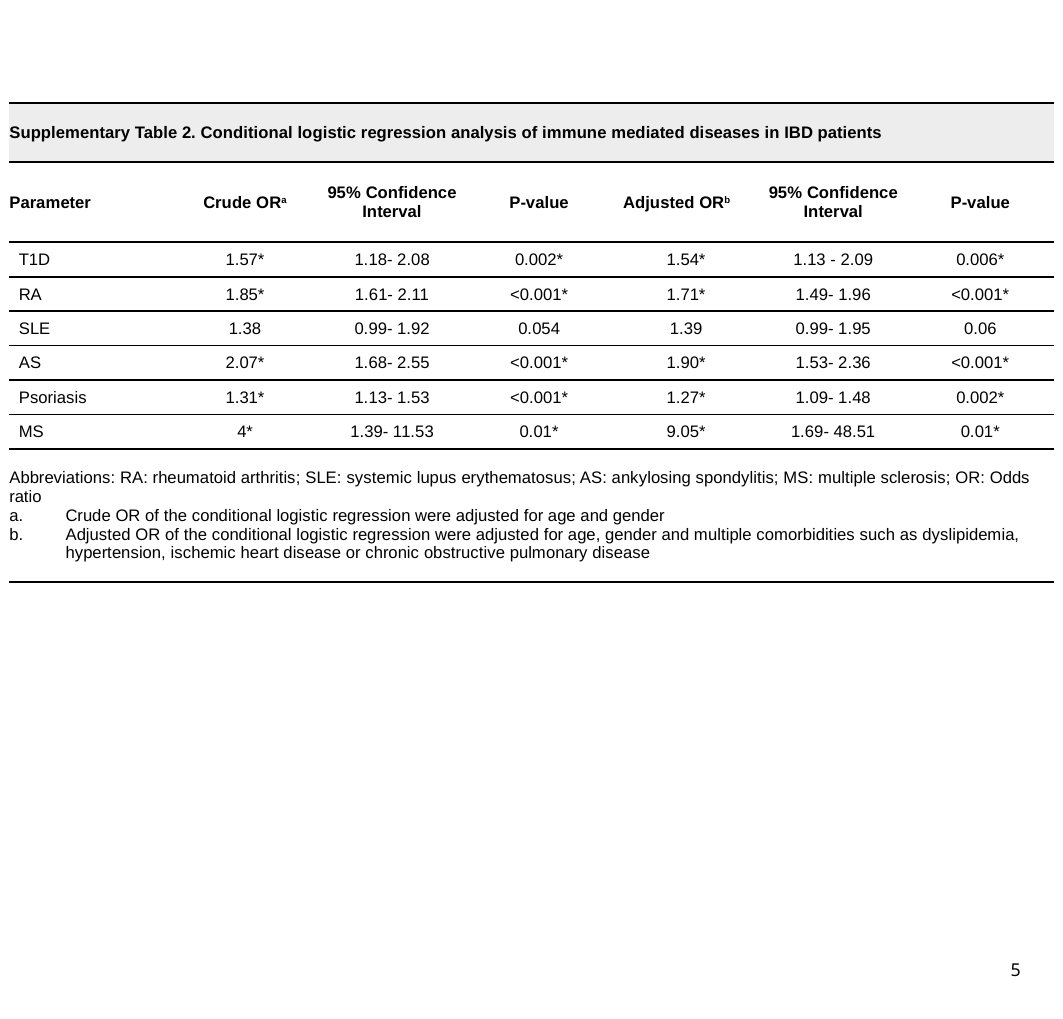

| Supplementary Table 2. Conditional logistic regression analysis of immune mediated diseases in IBD patients | | | | | | |
| --- | --- | --- | --- | --- | --- | --- |
| Parameter | Crude ORa | 95% Confidence Interval | P-value | Adjusted ORb | 95% Confidence Interval | P-value |
| T1D | 1.57\* | 1.18- 2.08 | 0.002\* | 1.54\* | 1.13 - 2.09 | 0.006\* |
| RA | 1.85\* | 1.61- 2.11 | <0.001\* | 1.71\* | 1.49- 1.96 | <0.001\* |
| SLE | 1.38 | 0.99- 1.92 | 0.054 | 1.39 | 0.99- 1.95 | 0.06 |
| AS | 2.07\* | 1.68- 2.55 | <0.001\* | 1.90\* | 1.53- 2.36 | <0.001\* |
| Psoriasis | 1.31\* | 1.13- 1.53 | <0.001\* | 1.27\* | 1.09- 1.48 | 0.002\* |
| MS | 4\* | 1.39- 11.53 | 0.01\* | 9.05\* | 1.69- 48.51 | 0.01\* |
| Abbreviations: RA: rheumatoid arthritis; SLE: systemic lupus erythematosus; AS: ankylosing spondylitis; MS: multiple sclerosis; OR: Odds ratio Crude OR of the conditional logistic regression were adjusted for age and gender Adjusted OR of the conditional logistic regression were adjusted for age, gender and multiple comorbidities such as dyslipidemia, hypertension, ischemic heart disease or chronic obstructive pulmonary disease | | | | | | |
5

## Slide 3
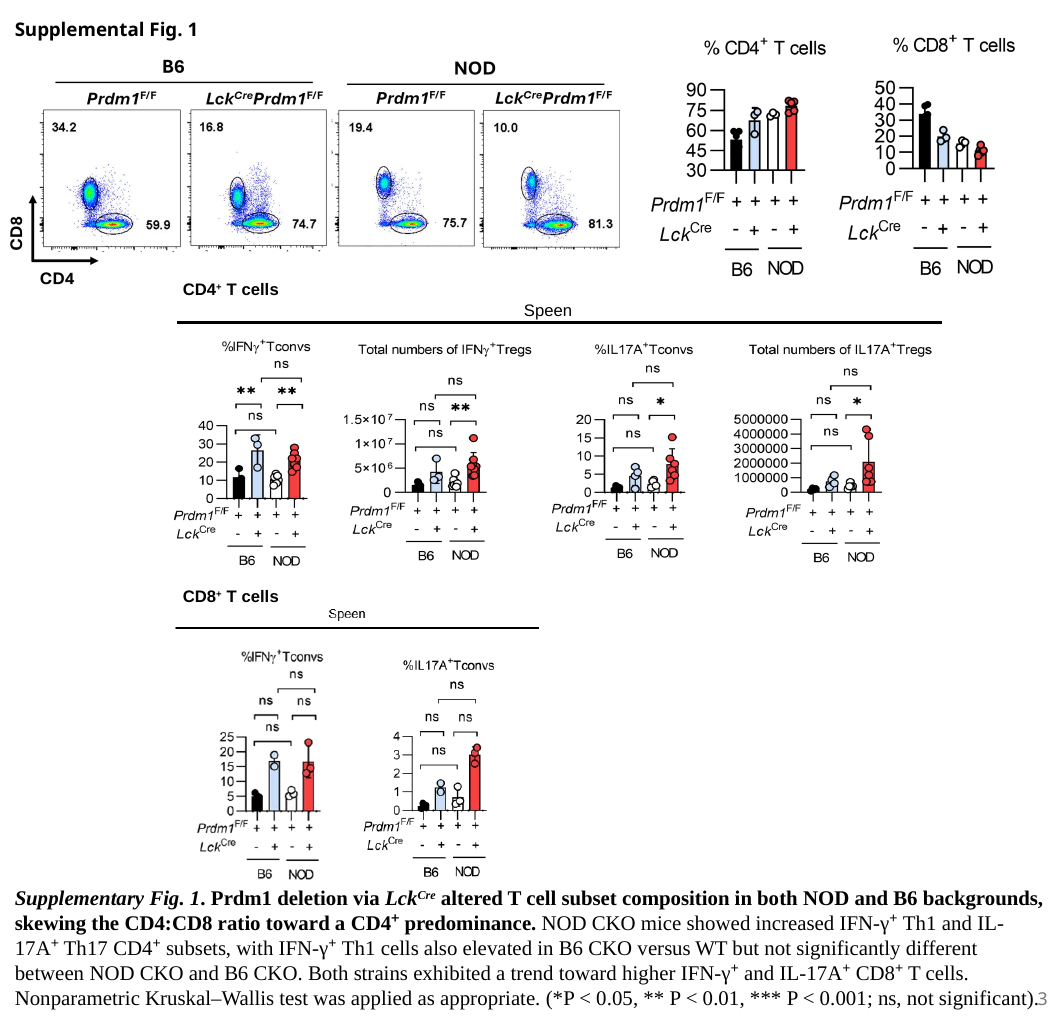

Supplemental Fig. 1
CD4+ T cells
Speen
CD8+ T cells
Supplementary Fig. 1. Prdm1 deletion via LckCre altered T cell subset composition in both NOD and B6 backgrounds, skewing the CD4:CD8 ratio toward a CD4⁺ predominance. NOD CKO mice showed increased IFN-γ⁺ Th1 and IL-17A⁺ Th17 CD4⁺ subsets, with IFN-γ⁺ Th1 cells also elevated in B6 CKO versus WT but not significantly different between NOD CKO and B6 CKO. Both strains exhibited a trend toward higher IFN-γ⁺ and IL-17A⁺ CD8⁺ T cells. Nonparametric Kruskal–Wallis test was applied as appropriate. (*P < 0.05, ** P < 0.01, *** P < 0.001; ns, not significant).
3

## Slide 4
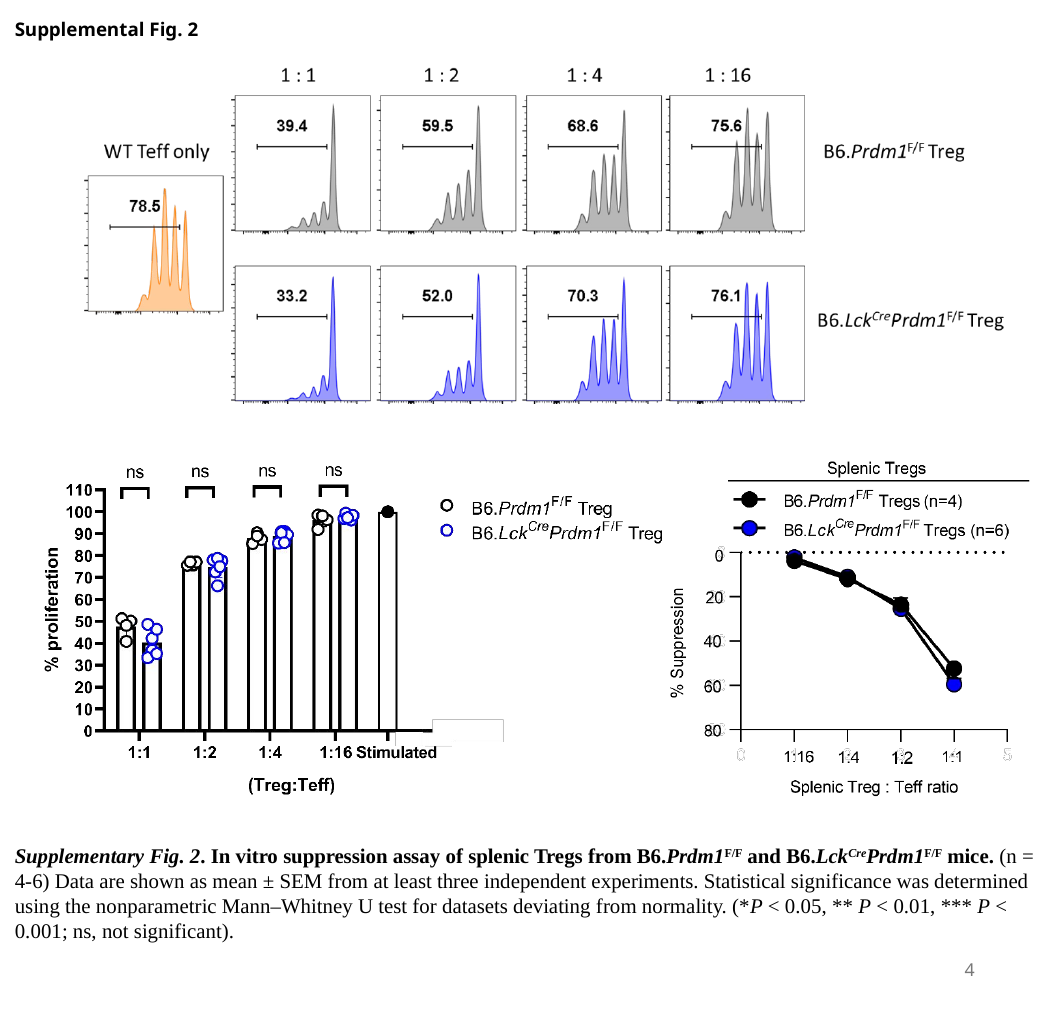

Supplemental Fig. 2
Supplementary Fig. 2. In vitro suppression assay of splenic Tregs from B6.Prdm1F/F and B6.LckCrePrdm1F/F mice. (n = 4-6) Data are shown as mean ± SEM from at least three independent experiments. Statistical significance was determined using the nonparametric Mann–Whitney U test for datasets deviating from normality. (*P < 0.05, ** P < 0.01, *** P < 0.001; ns, not significant).
4

## Slide 5
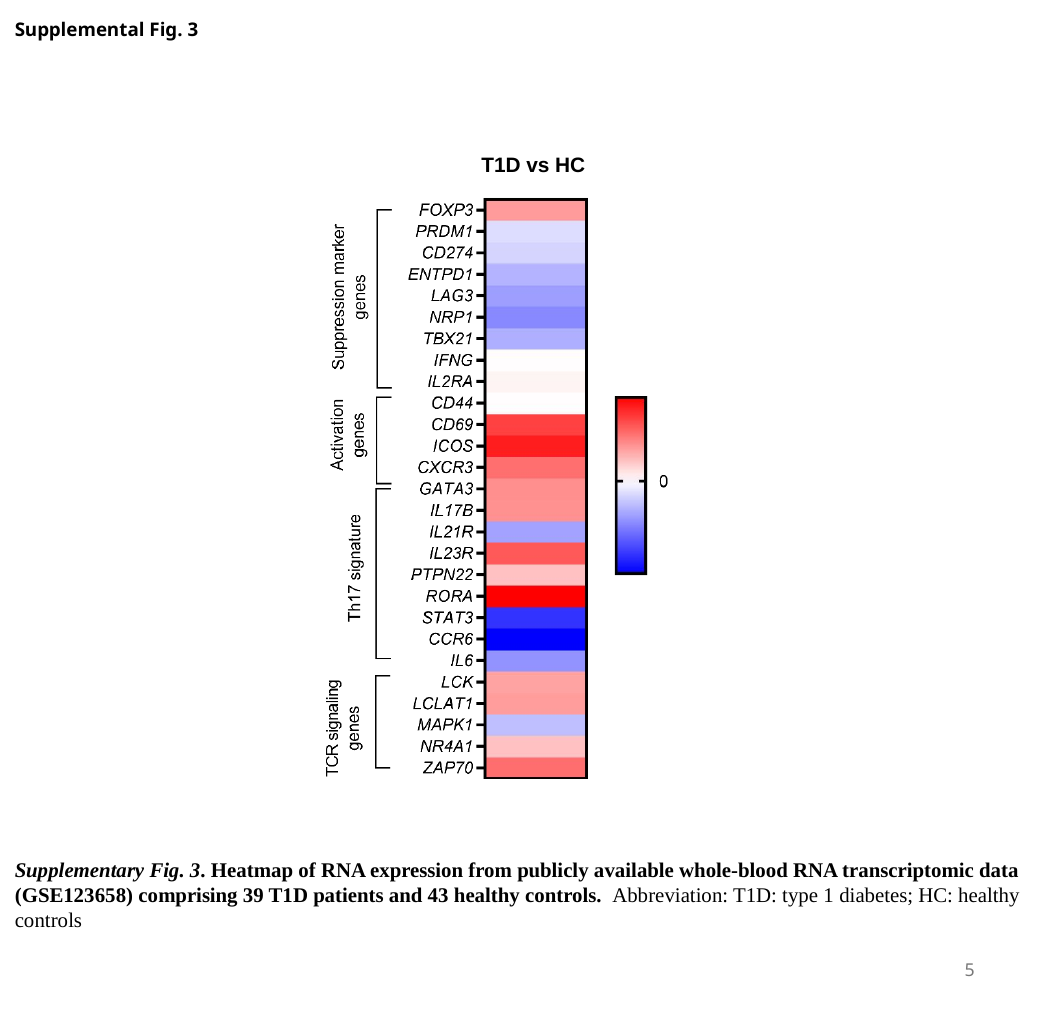

Supplemental Fig. 3
T1D vs HC
Supplementary Fig. 3. Heatmap of RNA expression from publicly available whole-blood RNA transcriptomic data (GSE123658) comprising 39 T1D patients and 43 healthy controls. Abbreviation: T1D: type 1 diabetes; HC: healthy controls
5
