# Supplementary material for: Blimp-1 benefits gut-homing regulatory T cells by maintaining migration/suppressive function in autoimmune diabetes-prone mice
Source: eBioMedicine. 2025 Nov 5;121:106002. doi: 10.1016/j.ebiom.2025.106002 (PMC12636379; doi:10.1016/j.ebiom.2025.106002)
Supplement: Supplementary Material [file mmc2.docx]

**Blimp-1 benefits gut-homing regulatory T cells by maintaining migration/suppressive function in autoimmune diabetes-prone mice**

Yi-Wen Tsai^1,2,3,4^, Yu-Wen Liu^5,6^, Chao-Yuan Hsu^5^, Shin-Huei Fu^5,6^, Ming-Wei Chien^5,6^, Jia-Ling Dong^7^, Chi-Chin Sun^4,8^, Chien-Tzung Chen^4,9^, Huey-Kang Sytwu^1,5,6,7*^

^1^ Graduate Institute of Medical Sciences, National Defense Medical University, Taipei, Taiwan ^2^ Department of Family Medicine, Chang Gung Memorial Hospital, Linkou, Taoyuan City, Taiwan ^3^ Department of Family Medicine, New Taipei Municipal Tucheng Hospital (Bulit and Operated by Chang Gung Medical Foundation), New Taipei City, Taiwan ^4^ School of Medicine, College of Medicine, Chang-Gung University, Taoyuan City, Taiwan ^5^ Department and Graduate Institute of Microbiology and Immunology, National Defense Medical University, Taipei, Taiwan ^6^National Institute of Infectious Diseases and Vaccinology, National Health Research Institutes, Zhunan, Miaoli, Taiwan ^7^Graduate Institute of Life Sciences, National Defense Medical University, Taipei, Taiwan ^8^Department of Ophthalmology, Chang Gung Memorial Hospital, Keelung, Taiwan ^9^Department of Plastic and Reconstruction Surgery, Chang Gung Memorial Hospital, Linkou, Taoyuan City, Taiwan

**Supplementary material and methods**

***Flow cytometric analysis***. Cells were isolated from spleen and mLN of mice. For surface marker staining, cells were labeled with fluorochrome-conjugated antibodies for murine CD4 (RM4-5,GK1.5), CD62L (MEL-14), CD44 (IM7), CD25 (PC61.5,PC61), ICOS (C398.4A), CD73(5’-NT), CTLA-4(UC10-4B9), PD-1(J43), LAG3(C987W), TIGIT(1G9), Nrp1(3E12), CD127(A7R34), CCR6(29-2L17), CD5(53-73) and CD69(H1.2F3). For detection of intracellular proteins, cells were stained for Foxp3 (FJK-16s), Nur77 (12.14), IFNγ (XMG1.2), ERK1/2 (pT202/Py204), S6 (Ps244), AKT (M89-61), Iκbα (XMG1.2) and Rorγt (AFKJS-9) using the Foxp3 Transcription Factor Staining Buffer Set (ThermoFisher). For cytokine production analysis, cells were first stimulated with phorbol 12-myristate 13-acetate (PMA) and ionomycin in the presence of monensin (all from Sigma, St. Louis, MO, USA) for 4-5 h prior to staining for Foxp3 (FJK-16s) and IFNγ (XMG1.2) using the same buffer set. All antibodies were purchased from eBioscience (San Diego, CA), BioLegend (San Diego, CA) or BD Pharmingen (San Diego, CA, USA). Flow cytometry was performed with a FACS Calibur (BD Pharmingen, San Jose, CA, USA). Flow Jo software (Tree Star, Ashland, OR, USA) was used for data analysis.

***RNA-seq*.** CD4^+^ T and Treg cells were freshly isolated from mice for transcriptome analysis. RNA-seq was performed on an Illumina HiSeq 2000 or Illumina NextSeq platform (Illumina). Sequencing was outsourced to Genomics (Taiwan) and data analysis was conducted using CLC Genomics Workbench version 8.0.1 (Qiagen).

***In vitro Treg suppression assay*.** CD4^+^CD25^+^ Tregs and CD4^+^CD25^-^ conventional T cells were isolated from spleens and mLNs of mice using the CD4^+^CD25^+^ Regulatory T Cell Isolation Kit (Miltenyi Biotec, 130-091-041). For *in vitro* suppression assays, Tregs were plated at a fixed number of 1 × 10^5^ cells per well in 96-well round plates and co-cultured with varying numbers of Tconv cells from *Prdm1^F/F^Lck^Cre^* NOD mice at different Treg:Tconv ratios. Cells were stimulated with Dynabeads™ Mouse T-Activator CD3/CD28 (Gibco, 11452D) and cultured for 72 hrs.

***Migration assay***. Migration assays were performed using 24-well Transwell plates with a 5.0 μm polycarbonate membrane (Corning Life Sciences, Corning, USA). Culture medium (150 μL) with or without 500 ng/mL of recombinant mouse CCL20 was placed in the lower chamber. Culture medium (350 μL) containing 2×10^6^ cells was placed in the upper chamber. After 3-3.5 h, migrated Foxp3^+^CD4^+^ Tregs in the lower chambers were calculated by the flow cytometry. Migration index was defined by dividing the number of the migrated cells in wells containing recombinant mouse CCL20 by the mean migration observed in the medium-only control wells [^1^](#_ENREF_1).

***Disease Monitoring and Histological Scoring.*** Mice were monitored twice weekly from 5 weeks of age for body weight, stool consistency, and rectal prolapse. For histological assessment, selected mice at 15 weeks of age were euthanized, and colons were collected for pathological evaluation. Formalin-fixed, paraffin-embedded sections were stained with hematoxylin and eosin (H&E) and scored independently by two blinded pathologists using a published system (Fu et al., Gut, 2015). Scoring parameters included mucosal architecture destruction (0–3), inflammatory cell infiltration (0–3), muscle thickening (0–3), and goblet cell depletion (0–1). Scores were summed to generate a total histological score.

***Adoptive transfer.*** Splenocytes were isolated from NOD.*Prdm1^F/F^Lck^Cre^* donor mice, and naïve T cells (CD4^+^CD25^-^CD62L^hi^) were purified using the Naïve CD4^+^ T Cell Isolation Kit (Miltenyi Biotec, 130-104-453). These naïve CD4^+^ T cells were intraperitoneally injected (1×10^6^/mouse) into NOD/SCID recipient mice. Two weeks post transfer, 1 × 10^6^ CD4^+^CD25^+^ Treg cells purified from control NOD.*Prdm1^F/F^* mice, NOD.*Prdm1^F/F^Lck^Cre^* or NOD.*Lck^Ptpn22^Lck^Cre^Prdm1^F/F^* mice were injected intraperitoneally into separate groups of these NOD/SCID mice that had previously received naïve CD4^+^ T cells.

**References**

1. Cook KW, Letley DP, Ingram RJ, et al. CCL20/CCR6-mediated migration of regulatory T cells to the Helicobacter pylori-infected human gastric mucosa. *Gut* 2014; **63**(10): 1550-9.
